# Phenotyping Root Systems in a Set of Japonica Rice Accessions: Can Structural Traits Predict the Response to Drought?

**DOI:** 10.1186/s12284-020-00404-5

**Published:** 2020-09-15

**Authors:** Paulo Henrique Ramos Guimarães, Isabela Pereira de Lima, Adriano Pereira de Castro, Anna Cristina Lanna, Patrícia Guimarães Santos Melo, Marcel de Raïssac

**Affiliations:** 1 Embrapa Arroz e Feijão, Rodovia GO-462, km 12, Santo Antônio de Goiás, GO 75375-000 Brazil; 2 Universidade Federal de Lavras, Departamento de Agricultura, Campus Universitário, Lavras, MG 37200-000 Brazil; 3 Universidade Federal de Goiás, Rodovia GO-462, km 0, Campus Samambaia, Goiânia, GO 74001-970 Brazil; 4 Univ Montpellier, CIRAD, INRA, Montpellier SupAgro, AGAP, Montpellier, France

**Keywords:** *Oryza sativa*, Root system architecture, PVC pipes, Genetic variation, Phenotypic plasticity

## Abstract

**Background:**

The root system plays a major role in plant growth and development and root system architecture is reported to be the main trait related to plant adaptation to drought. However, phenotyping root systems in situ is not suited to high-throughput methods, leading to the development of non-destructive methods for evaluations in more or less controlled root environments. This study used a root phenotyping platform with a panel of 20 japonica rice accessions in order to: (i) assess their genetic diversity for a set of structural and morphological root traits and classify the different types; (ii) analyze the plastic response of their root system to a water deficit at reproductive phase and (iii) explore the ability of the platform for high-throughput phenotyping of root structure and morphology.

**Results:**

High variability for the studied root traits was found in the reduced set of accessions. Using eight selected traits under irrigated conditions, five root clusters were found that differed in root thickness, branching index and the pattern of fine and thick root distribution along the profile. When water deficit occurred at reproductive phase, some accessions significantly reduced root growth compared to the irrigated treatment, while others stimulated it. It was found that root cluster, as defined under irrigated conditions, could not predict the plastic response of roots under drought.

**Conclusions:**

This study revealed the possibility of reconstructing the structure of root systems from scanned images. It was thus possible to significantly class root systems according to simple structural traits, opening up the way for using such a platform for medium to high-throughput phenotyping. The study also highlighted the uncoupling between root structures under non-limiting water conditions and their response to drought.

## Background

The root system plays a major role in whole plant growth and development: it is the well-known “hidden half”, as described by Eshel and Beeckman (2013). Roots are directly involved in plant health, growth and survival, through water and nutrient uptake (Zhu et al. 2011; Takehisa et al. 2012; Sozzani and Iyer-Pascuzzi 2014). They are also the place where hormone synthesis and consumption take place, acting on whole plant hormonal regulation (Zhang et al. 2017; Atia et al. 2018; Ramireddy et al. 2018). In relation to drought, the root system is responsible for the avoidance mechanism that maintains water uptake and thus favorable organ water status under conditions of limited soil water. The architecture and morphological plasticity of a root system under drought are considered to be key traits driving the adaptive response of plants to water deficit (Henry 2013; Brunner et al. 2015; Muthurajan et al. 2018; Bristiel et al. 2019; Chaichi et al. 2019), expecting a direct impact on maintenance of grain yield. Nevertheless, the link between root traits and the maintenance of grain yield under drought is complex and needs further investigation before it can be used directly in breeding programs (Dorlodot et al. 2007; Kondo et al. 2003; Gowda et al. 2011; Comas et al. 2013; Han et al. 2016; Li et al. 2017).

When considering drought, the first information needed concerns the inter and intra-annual probability of water deficit occurrence, its severity and duration and its timing with rice phenological stages (Heinemann et al. 2008). Under a severe water deficit, the dynamics of root system growth are a key factor for plant adaptation (Matthews et al. 1984), as well as its plasticity, which is the ability to modify its growth and structure in varying environments (Price et al. 2002a). Changes in plant root system architecture may allow the selection of an ideal root system for different environments, with better nutrient uptake capacity, which would allow higher yield levels even under adverse weather conditions (Lynch 2007).

Due to their belowground growth, roots have long remained the principal challenge to phenotyping. In recent times, agronomists and breeders have attempted to characterize the development of root structure, morphology and dynamics (Henry 2013; Paez-Garcia et al. 2015; Cendrero-Mateo et al. 2017; Bray and Topp 2018). Some studies have been focused on the relationship between morphological traits, anatomical functions and root developmental processes (Lynch 2007, 2014; Gu et al. 2017; Passot et al. 2018). The search for root traits conferring high efficiency in resource uptake, mainly in nutrient and water use efficiencies (NUE and WUE), has been stepped up in rice breeding programs (Bernier et al. 2008; Han et al. 2016; Araus et al. 2018; Mir et al. 2019), especially in upland environments where short dry spells are common in the central Brazilian plateau (Guimarães et al. 2011; Terra et al. 2013).

The mechanisms that control root system structure and morphology conferring greater tolerance to drought still remain uncertain (Asch et al. 2005; Liu et al. 2006). In rice there is a high diversity of root morphological traits that is enhanced in response to drought (Kondo et al. 2003; Gowda et al. 2011). In order to use it in breeding programs for drought tolerance improvement, breeders established a set of root traits such as: root length, root thickness, root density, root branching, root length density, rooting depth, surface area, root diameter and the distribution of root biomass in the soil profile (Price et al. 2002a; Matsui and Singh 2003; Kondo et al. 2003; Ganapathy et al. 2010; Henry et al. 2012; Kano-Nakata et al. 2013; Kuijken et al. 2015). Nevertheless, these traits do not give a global view on the root system and do not allow to decipher what is the influence of each one in maintaining grain yield under drought (Gowda et al. 2011; Mickelbart et al. 2015; Kadam et al. 2017).

Intensive genetic studies have been led to the identification of numerous *QTLs* and the relative genes underlying the genetic control of different root traits in rice (Chen et al. 2013; Uga et al. 2015; Han et al. 2018; Ramanathan et al. 2018; He et al. 2019 ; Sandhu et al. 2019). However, phenotyping of root structural traits is difficult and laborious to implement and constitutes the main bottleneck in using genomics approaches. Under field or pot conditions, the complete excavation of a root system is not realistic, as root system removal is time-consuming and destructive, with risks of root structure loss (Masuka et al. 2012; Armengaud et al. 2009; Zhu et al. 2011; Wasson et al. 2012). In order to access better the complete root system, in recent years, different approaches have been used, such as PVC pipes (Shashidhar et al. 2012; Guimarães et al. 2011), hydroponic systems (Courtois et al. 2013) semi-hydroponic systems (Chen et al. 2017) or rhizotrons (Price et al. 2012; Shrestha et al. 2014). These phenotyping approaches are based on scanning and analyzing plant images. It is thus possible to obtain a large number of images in a relatively short time, increasing phenotyping capacity, enabling greater accuracy and leading to increased breeding efficiency (Pratap et al. 2019). Up to now, these methods do not describe the structure of the root system, which conditions its spatial organization within the soil profile (explored soil volume through root types, branching levels and elongation rates) and extent of the contact and interactions between the plant and the rhizosphere (Lynch 1995; Bates and Lynch 2000; Gilroy and Jones 2000; Leitner et al. 2010; Orman-Ligeza et al. 2014). According to Orman-Ligeza et al. (2014), the knowledge about the root system structure have the potential to support a second green revolution targeting crop performance under water and nutrient restriction.

This study used a root phenotyping platform with a panel of 20 japonica rice accessions in order to: (i) assess their genetic diversity for a set of structural and morphological root traits and classify the different types; (ii) analyze the plastic response of their root system to a water deficit at reproductive phase and (iii) explore the ability of the platform for high-throughput phenotyping of root structure and morphology.

## Material and Methods

### Plant Material

A rice diversity panel consisting of 217 accessions of tropical japonica rice was obtained from the International Rice Research Institute - IRRI and purified through single seed descent method, prior to phenotyping. Twenty accessions were selected (Supplementary Fig. S1), with more or less the same growth cycle. These accessions were then phenotyped in the greenhouse.

### Experimental Conditions

**Fig. 1:**
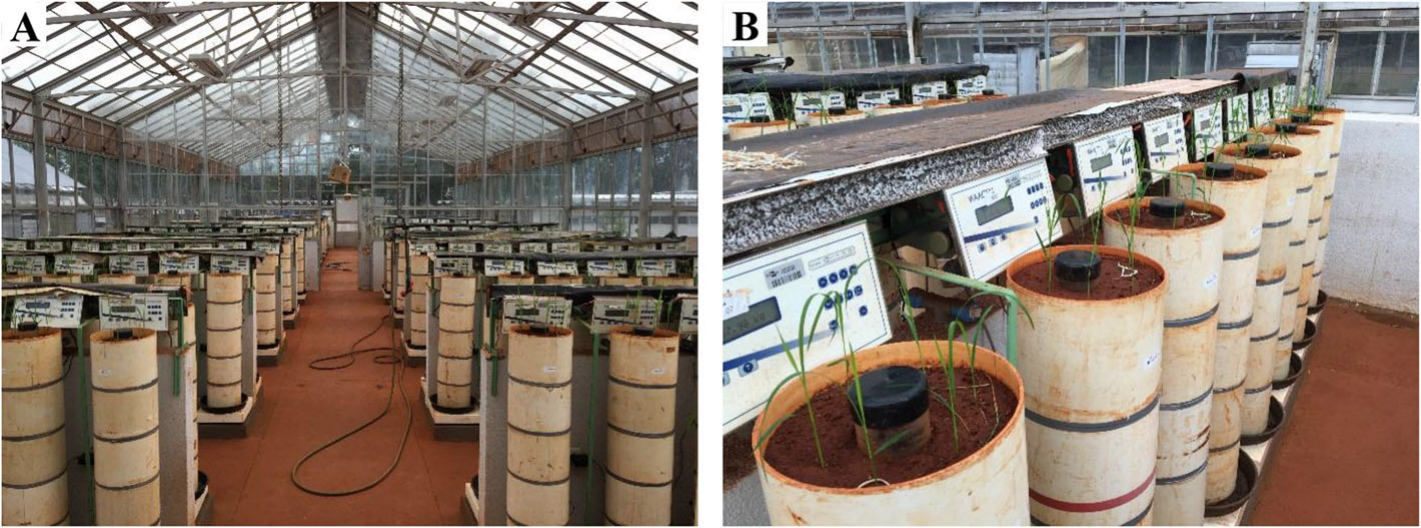
Experiment in greenhouse. *View of SITIS phenotyping platform* (**a**), *view of the PVC pipes containing the soil, the transparent acrylic tube in the middle and the rice seedlings around it, before thinning to three plants* (**b**)

This experiment was conducted in a plant phenotyping platform facility called the *Integrated System for Drought-Induced Treatment* (acronym SITIS, in Portuguese) from September 2015 to January 2016, at Embrapa Rice and Beans, in Santo Antônio de Goiás, GO, Brazil (16°27′28″S, 49°19′52″W, at 823 m above sea level). The facility provided 384 soil columns, packed in PVC pipes 0.25 m in diameter and 1.00 m in height, placed on digital scales with an irrigation point for each pipe (Fig. 1). The amount of water used by the plants was monitored in each pipe by the difference in weight. During the experimental period, the air temperature and relative humidity fluctuated from 17.4 °C to 37.6 °C and 7.7 to 81.5% respectively (Supplementary Fig. S2), in relation to a typical drought spell in the region, called the “veranico”, a period of 10–15 days without rain during the crop season (De Datta 1981; Faleiro and Farias Neto 2008).

The experiment was carried out in a randomized block design with two water treatments and three replicates, using 120 soil columns (60 pipes per treatment). The soil was sieved (125 mm mesh) to remove the larger aggregates and enriched with minerals, including 4 g of 04–14-08 (NPK) fertilizer. Fertilizer was applied 2 days before the sowing day and no fertilization was performed during the plant cycle. The pipes contained around 51.3 kg of soil (dry weight) sampled at a depth of between 40 and 50 cm in a field at the Capivara experimental site belonging to Embrapa Rice and Beans. The soil was characterized by a field capacity (FC) of 28.9% and a wilting point (WP) of 17.1% moisture content (in g water/g dry soil). The sowing was performed in excess on September 2015 in order to ensure the ultimate presence of three plants per pipe, according to the germination test. At 12 days after sowing, a thinning was carried out to leave only three plants per pipe. Lastly, three accessions were not adapted to the photoperiod and climate conditions and extended their reproductive phase up to 100 days, lagging well behind the other 17 accessions, so they were removed from the statistical analysis.

### Water Treatments

In the irrigated treatment, the soil columns were adjusted daily to *FTSW* =0.8 (Fraction of Transpirable Soil Water), which was calculated separately for each pipe. The water was applied to the top of the pipe, at the end of the afternoon. The *FTSW* was calculated according to Soltani et al. (2000):

$$ FTSW=\frac{ATSW}{TTSW}=\frac{W_t-{W}_f}{W_i-{W}_f} $$

where,

*ATSW*: is the available transpirable soil water determined for each soil column as the pipe weight on a specific day (*W*_*t*_) minus the final pipe weight (*W*_*f*_), i.e., pipe weight when the daily transpiration rate decreased to < 0.2 of well-watered plants.

*TTSW*: is the total transpirable soil water calculated for each treatment as the difference between the initial and final pipe weights (*W*_*i*_ and *W*_*f*_, respectively).

In the water deficit treatment, the soil columns were adjusted daily to *FTSW* =0.8 up to 10 days after panicle initiation (*PI*), and then adjusted to *FTSW* =0.4 (moderate water deficit) up to the heading of plants in the irrigated treatment. In order to trigger the water deficit at exactly 10 days after *PI* in each accession, we used the average value of three previous trials conducted with the same accessions in the field (Porangatu, Brazil, in 2013 and 2014) and in a greenhouse (Santo Antônio de Goiás, Brazil, in 2014). We estimated the date of panicle initiation as corresponding to five phyllochrons (Matsushima 1966; Nemoto et al. 1995) and occurring around 35 days before flowering (Matsushima 1966; De Datta 1981). Then, we gathered the genotypes in 4 groups of accessions having the same earliness and we triggered the water deficit treatment in the same day for the accessions within a group at the estimated date of panicle initiation (*PI*) = 10 days. Irrigation in the “drought” treatment was also controlled daily, at the end of the afternoon, by adding water to the tray placed under each pipe. The water was added to the tray because the roots of all the accessions had reached the bottom of the column, so it could be assumed that the whole soil volume had been colonized by the roots.

The end of the drought treatment was determined in each accession by panicle emergence in the irrigated treatment. At this stage, the shoot of one plant per pipe was collected and dried in oven at 70 °C for 72 h, in order to determine the shoot dry weight. The two remaining plants in the PVC pipe were conducted at full irrigation up to harvest, in order to analyze their yield. Unfortunately, the daily maximum temperature (*T*_*max*_) oscillated between 30 and 35 °C leading to high spikelet sterility in some accessions adapted to altitude or temperate climates. We did not use hereafter the data on these plants.

### Root Measurements

Root measurements were performed once in the experiment on all the pipes, at the end of the water deficit, when panicles emerged on the plants of the irrigated treatment. The root system was assessed by taking root images through acrylic tubes of 6.4 cm diameter and 67 cm height installed in the middle of the PVC pipes. Root images were taken at a depth of 0 to 20 cm (20 cm layer), 20 to 40 cm (40 cm layer) and 40 to 60 cm (60 cm layer). The acrylic tubes were covered by a cap to avoid any entry of soil, water or organic waste. The images were taken with a CI - 600 Cano Scan scanner (CID Bio - Science, Version 3.1.19). For a more accurate root length and root diameter, the images obtained with the scanner were divided into ten sub-images prior to analysis (Tajima and Kato 2013) with ImageJ software (Rasband 2011), followed by automatic image processing with WinRhizo software (Regent Instruments Inc 2016). With WinRhizo software, the roots were partitioned into 10 diameter classes in 0.5 mm (0 mm - 4.5 mm) steps and root lengths for each root diameter class were computed. The root length (*RL*), diameter (*RD*) and volume (*RV*) were determined by WinRhizo software. We calculated the total root volume (*TOT*_*VOL*_) and total root length (*TOT*_*RL*_) by adding the root volume and root length respectively found in each analyzed layer. It must be stressed that the only roots that are measured are the ones being in contact with the acrylic tube and appearing on the scanned image.

According to previous works on *Picea glauca* (Bauhus and Messier 1999) and in rice (Price et al. 1989; Dien et al. 2017), we divided the roots into two diameter classes: 1) fine roots with a diameter ≤ 0.5 mm; and 2) thick roots with 1.0 mm ≤ diameter ≤ 2.5 mm, and we calculated the length of each root type (fine and thick roots respectively). In this study, we considered thick roots as primary roots and fine roots as secondary or tertiary ones. As the accessions have distinct diameters of primary or secondary roots, we opted to suppress the 0.5–1.0 mm class to avoid any mistake in root classification. The lacking root length values (with a diameter between 0.5 and 1.0 mm) were taken into account for the total root length in each layer. We then calculated some derived traits as listed below:

- Coefficient of maintenance for fine roots (${\upalpha}_{FR{L}_n}$):

$$ {\upalpha}_{FR{L}_n}=\frac{FR{L}_{n+1}}{FR{L}_n}, $$

where

*n*: is the number of the layer, with

*n*= 1 for 0–20 cm layer, *n* = 2 for 20–40 cm layer and *n* = 3 for the 40–60 cm layer.

- Coefficient of maintenance for thick roots (${\upalpha}_{TR{L}_n}$):

$$ {\upalpha}_{TR{L}_n}=\frac{TR{L}_{n+1}}{TR{L}_n} $$

- Branching index (*BI*):

$$ {BI}_n=\frac{FR{L}_n}{TR{L}_n} $$

- Thick root diameter (*DIAM* _ *TR*_*n*_):

$$ DIAM\_{TR}_n=\frac{\left(1.25\ x\ {RL}_1\ \right)+\left(1.75\ x\ {RL}_2\ \right)+\left(2.25\ x\ {RL}_3\right)}{RL_1+{RL}_2+\kern0.5em {RL}_3}, $$

where

*RL*_1_: is the root length for the root diameter class between 1.0 and 1.5 mm.

*RL*_2_: is the root length for the root diameter class between 1.5 and 2.0 mm.

*RL*_3_: is the root length for the root diameter class between 2.0 and 2.5 mm.

- Thick root diameter reduction (${RED}_{T{R}_{20_{60}}}$): the hypothesis was put forward here that a low reduction in primary root diameter between 20 and 60 cm, together with a high *DIAM* _ *TR*_60_, was a good predictor of the potential maximum depth of the rooting system. Then ${RED}_{T{R}_{20_{60}}}$ was calculated as:
  $$ {RED}_{T{R}_{20_{60}}}=\left(\frac{DIAM\_{TR}_{20}- DIAM\_{TR}_{60}}{DIAM\_{TR}_{20}}\right)\ x\ 100 $$

where,

*DIAM* _ *TR*_20_: is the thick root diameter at 20 cm.

*DIAM* _ *TR*_60_: is the thick root diameter at 60 cm.

Root trait data in the upper 0–20 cm was considered the “topsoil” section and the sections 20–40 cm and 40–60 cm were considered the “subsoil” section. Descriptions and abbreviations of the 29 traits (28 root traits, and 1 shoot trait) are presented in Table 1.

**Table 1:** Description of measured traits in the tropical japonica rice panel grown in greenhouse

Trait	Abbreviation	Description	Unit
Root length at 20 cm	*RL*_20_	Total root length in the 0 to 20 cm layer	cm
Root length at 40 cm	*RL*_40_	Total root length in the 20 to 40 cm layer	cm
Root length at 60 cm	*RL*_60_	Total root length in the 40 to 60 cm layer	cm
Root volume at 20 cm	*RV*_20_	Total root volume in the 0 to 20 cm layer	cm^3^
Root volume at 40 cm	*RV*_40_	Total root volume in the 20 to 40 cm layer	cm^3^
Root volume at 60 cm	*RV*_60_	Total root volume in the 40 to 60 cm layer	cm^3^
Root diameter at 20 cm	*RD*_20_	Average root diameter in the 0 to 20 cm layer	mm
Root diameter at 40 cm	*RD*_40_	Average root diameter in the 20 to 40 cm layer	mm
Root diameter at 60 cm	*RD*_60_	Average root diameter in the 40 to 60 cm layer	mm
Fine root length at 20 cm	*FRL*_20_	Total root length with diameter ≤ 0.5 mm at 20 cm	cm
Fine root length at 40 cm	*FRL*_40_	Total root length with diameter ≤ 0.5 mm at 40 cm	cm
Fine root length at 60 cm	*FRL*_60_	Total root length with diameter ≤ 0.5 mm at 60 cm	cm
Thick root length at 20 cm	*TRL*_20_	Total root length with 1.0 mm ≤ diameter ≤ 2.5 mm at 20 cm	cm
Thick root length at 40 cm	*TRL*_40_	Total root length with 1.0 mm ≤ diameter ≤ 2.5 mm at 40 cm	cm
Thick root length at 60 cm	*TRL*_60_	Total root length with 1.0 mm ≤ diameter ≤ 2.5 mm at 60 cm	cm
Maintenance coefficient of fine roots between 20 and 40 cm	${\upalpha}_{FR{L}_{20\_40}}$	Ratio between fine root length at 40 cm and fine root length at 20 cm	–
Maintenance coefficient of fine roots between 40 and 60 cm	${\upalpha}_{FR{L}_{40\_60}}$	Ratio between fine root length at 60 cm and fine root length at 40 cm	–
Maintenance coefficient of thick roots between 20 and 40 cm	${\upalpha}_{TR{L}_{20\_40}}$	Ratio between thick root length at 40 cm and thick root length at 20 cm	–
Maintenance coefficient of thick roots between 40 and 60 cm	${\upalpha}_{TR{L}_{40\_60}}$	Ratio between thick root length at 60 cm and thick root length at 40 cm	–
Branching index at 20 cm	*BI*_20_	Ratio between fine root length at 20 cm and thick root length at 20 cm	–
Branching index at 40 cm	*BI*_40_	Ratio between fine root length at 40 cm and thick root length at 40 cm	–
Branching index at 60 cm	*BI*_60_	Ratio between fine root length at 60 cm and thick root length at 60 cm	–
Total root length	*TOT*_*RL*_	Sum of root lengths at 20 cm, 40 cm and 60 cm	cm
Total root volume	*TOT*_*VOL*_	Sum of root volumes at 20 cm, 40 cm and 60 cm	cm^3^
Thick root diameter at 20 cm	*DIAM* _ *TR*_20_	Thick root diameter at 20 cm	mm
Thick root diameter at 40 cm	*DIAM* _ *TR*_40_	Thick root diameter at 40 cm	mm
Thick root diameter at 60 cm	*DIAM* _ *TR*_60_	Thick root diameter at 60 cm	mm
Thick root diameter reduction between 20 and 60 cm	${RED}_{T{R}_{20_{60}}}$	Difference ratio between thick root diameters between 20 and 60 cm	%
Shoot dry weight	*SDW*	Shoot biomass of one plant target	g plant^-1^

### Statistical Data Analysis

The experimental design was a randomized block, in a factorial arrangement, with the accessions and water treatment as factors. The analysis of variance (ANOVA) was performed to evaluate whether or not the ANOVA assumptions were met. After these preliminary evaluations, a joint variance analysis was performed for all the traits (those estimated by WinRhizo and the derived traits) within each layer as follow:

$$ {Y}_{ikl}=\mu +{\rho}_{k/l}+{\beta}_i+{\delta}_l+{\beta \delta}_{il}+{\varepsilon}_{ikl} $$

where,

*Y*_*ikl*_: is the observed value of *i* ^*th*^ accession, in the *l* ^*th*^ water treatment in *k* ^*th*^ block.

*μ*: is the constant inherent to all observations.

*ρ*_*k*/*l*_: is the effect of the *k* ^*th*^ block within the *l* ^*th*^ water treatment.

*β*_*i*_: is the effect of the *i* ^*th*^ accession.

*δ*_*l*_: is the effect of the *l* ^*th*^ water treatment.

*βδ*_*il*_: is the effect of the interaction between the *i* ^*th*^ accession and *l* ^*th*^ water treatment.

*ε*_*ikl*_: is the effect of the experimental error associated with the *ikl* ^*th*^ plot, assuming *ε*_*ikl*_ *NID* ∩ (0, *σ*^2^).

### Method to Selecting Traits for Root System Characterization Under Irrigated Conditions

General correlations between the different traits were examined using Pearson correlation coefficients. The correlation matrix between the different traits was obtained with the *psych* package (Revelle 2017). Root traits with a low correlation coefficient (*r*^2^) and a biological importance in the irrigated treatment were chosen. A Principal Component Analysis (PCA) and a Hierarchical Cluster Analysis (HCA) were used as a quantitative and independent approaches to identify determinants of root architecture variability across accessions. The PCA and HCA were carried out with the *FactoMineR* package (Lê et al. 2008). The approach used to carry out the PCA in *FactoMineR* is described in detail by Lê et al. (2008). For the purposes of this study, *TOT*_*VOL*_ and the geographic origin were entered as supplementary variables. The *factoextra* package was used to visualize the output of the PCA analysis (Kassambara and Mundt 2017). In addition to the HCA and PCA, Pearson correlations were used to find the interrelationship between the chosen root traits. The HCA was performed using a combination of Ward’s linkage method (Ward 1963), adopting the Euclidean distances as a measure of dissimilarity. The different clusters found were tested by Tukey’s Honest (HSD) test (*P <* 0.05).

### Index to Assess Water Deficit Impact on Root System Development

The response to drought (*VAR∆*_*DC*/*C*_) for each selected trait was calculated and adapted according to the equation described by Rebolledo et al. (2012):

$$ VAR{\Delta }_{DC/C}=\frac{Y_{ik}-{\overline{Y}}_{i.}}{{\overline{Y}}_{i.}} $$

where,

*Y*_*ik*_: is the value found for accession *i* in block *k* under drought conditions.

${\overline{Y}}_{i.}$: is the average for accession *i* in the *k*^*th*^ block under irrigated conditions. According to this, a negative value of *VAR∆*_*DC*/*C*_ corresponds to a reduction by the drought treatment.

The total volume reduction of the root system was used as a comprehensive trait of root system growth and used as the main classificatory trait in response to drought. The *ggplot2* package was used to construct box-plots to visualize the variability between the different clusters (Wickham 2009). The different clusters and response groups (RG) found were tested by Tukey’s Honest (HSD) test (*P* < 0.05). The analysis was carried out with R software (R Core Team 2017).

## Results

### Root Phenotyping

Table 2 presents a summary of the results obtained for the overall root phenotyping and includes the two-way ANOVA. The accessions displayed large variations in the evaluated root traits across the different layers and under the two water treatments (Table 2). The distribution of the root traits was normal overall, with the exception of $\kern0.75em {\upalpha}_{FR{L}_{20\_40}}$, which needed to be transformed by Box-Cox transformation (Box and Cox 1964). As commonly found in root studies, the coefficients of variation (CVs) displayed high values, since they cumulated variations due to genetic diversity, the water treatments and inter-replicate variability. As expected, the diameters displayed very low variability, both for the average root diameter (*RD*) and thick root diameter (*DIAM* _ *TR*), with their CVs ranging from 8.2 to 10.5% and 1.7 to 2.8%, respectively, whatever the layer considered (Table 2). On the other hand, the branching index (*BI*) was highly variable between accessions and water treatments (CV between 34.7 and 55.4% depending on the layer). A general observation was also the increase in CV with depth for all variables, leading to less significant differences between accessions and/or treatments in the 40–60 cm depth layer than in the superficial layer.

**Table 2:** Descriptive statistics of all measured traits in the japonica rice panel and significance of Anova

Trait	TRT	Accessions (A)	Block/TRT	A x TRT	CV(%)	Minimum	Maximum	Mean
*RL*_20_	*	**	ns	**	25.32	1,158	11,363	4,894
*RD*_20_	**	**	ns	**	8.17	0.38	0.68	0.50
*RV*_20_	ns	**	ns	**	22.30	2.88	15.11	8.30
*FRL*_20_	**	**	ns	**	28.83	659.8	9,259.3	3,723.20
*TRL*_20_	ns	**	ns	**	27.78	70.18	549.73	285.64
*BI*_20_	**	**	ns	ns	34.78	4.63	29.61	13.32
*DIAM* _ *TR*_20_	ns	**	ns	**	1.72	1.33	1.53	1.44
*RL*_40_	ns	**	ns	ns	27.02	1,206	7,047	3,432
*RD*_40_	ns	**	ns	ns	9.50	0.38	0.65	0.51
*RV*_40_	ns	ns	ns	**	23.39	2.25	10.65	6.43
*FRL*_40_	ns	**	ns	ns	34.94	630.1	5,859.5	2,389
*TRL*_40_	**	ns	ns	*	36.58	41.29	383.28	187.18
*BI*_40_	**	*	ns	ns	49.52	3.95	45.89	14.72
*DIAM* _ *TR*_40_	*	**	ns	ns	2.65	1.32	1.48	1.40
*RL*_60_	ns	*	ns	ns	28.15	1,341	6,075	2,987
*RD*_60_	ns	**	ns	ns	10.46	0.41	0.67	0.51
*RV*_60_	ns	ns	ns	ns	27.29	2.55	11.65	5.91
*FRL*_60_	ns	**	ns	ns	37.73	586.9	5,002.6	1,997.70
*TRL*_60_	ns	ns	ns	ns	49.43	18.76	397.19	156.95
*BI*_60_	*	**	ns	ns	55.41	3.49	63.58	15.75
*DIAM* _ *TR*_60_	ns	**	*	ns	2.80	1.27	1.50	1.38
*TOT*_*RL*_	ns	**	ns	*	21.97	4,965	22,588	11,312
*TOT*_*VOL*_	ns	**	ns	**	17.48	10.98	32.09	20.63
${RED}_{T{R}_{20_{60}}}$	ns	ns	*	*	72.76	-4.18	12.47	3.96
${\upalpha}_{FR{L}_{20\_40}}$	ns	**	ns	*	35.18	0.15	2.40	0.75
${\upalpha}_{FR{L}_{40\_60}}$	*	**	*	**	23.41	0.32	1.62	0.87
${\upalpha}_{TR{L}_{20\_40}}$	ns	*	ns	*	37.01	0.12	1.98	0.70
${\upalpha}_{TR{L}_{40\_60}}$	**	ns	**	ns	40.36	0.12	2.87	0.88
*SDW*	**	**	ns	ns	17.07	8.98	53.51	21.51

At 20 cm (the most discriminant layer), it was found that all the traits displayed significant interaction effects, except *BI*_20_ (Table 2): this means that the accessions had different responses to drought, whatever their own root structure and development at the time of heading. This phenomenon was found in the superficial layer, and was less significant in the deeper layers, probably because of the duration of water deficit, which lasted between 20 and 30 days depending on the accession, and because new emerging primary roots in the superficial layer did not have time to colonize the deeper layer.

As also expected, morphological traits decreased with depth, because of the gradual colonization of deep layers by the growing root system: root length (*RL*), thick and fine root length (*TRL* and *FRL*), and root volume (*RV*) had continuously decreasing values from the superficial layer to the 40 and 60 cm layers. Interestingly, the branching index (*BI*) was very variable within a layer, but it remained quite constant through the layers: the ability of a bearer root to generate borne roots that elongated could be considered as a constant genetic parameter, at least under given soil and climate conditions (Table 2). *RD*, which was given by the WinRhizo software, was no longer considered in this study, as it was a mean value on all root types, without clear morphological meaning.

Phenotyping the root development and morphology of a set of accessions with two water treatments could not be carried out using all these traits. In order to select a simple, reduced set of traits, it was necessary to understand the linkage existing between them, in order to eliminate “duplicate traits” and to keep the most representative and biologically significant ones. Supplementary Table S1 gives the correlations between all the traits.

Within a layer, high correlations were recorded between thick root length (*TRL*) and root volume (*RV*): *r* = 0.96, 0.90 and 0.89 at 20, 40 and 60 cm respectively. Moreover, whatever the layer, *TRL* was highly correlated to the total root volume (*TOT*_*VOL*_) (at 0.93, 0.92 and 0.71 top down), including thick, intermediate and fine roots of all the layers. Complementarily, fine root length (*FRL*) was highly correlated to total root length (*RL*): *r* = 0.99, 0.98 and 0.98 in the three layers respectively (Supplementary Table S1) and to the plant total root length (*TOT*_*RL*_) *r* = 0.93. Thus, *TRL* appeared to be a good proxy of total root volume, while *FRL* was a good proxy of total root length. In addition, considering the homogeneity of root density in annual crops, *TOT*_*VOL*_ can be considered as representative of root biomass.

Between the three layers, *TRL* was well preserved: *TRL*_20_ was correlated to *TRL*_40_ at *r* = 0.77, *TRL*_40_ to *TRL*_60_ at *r* = 0.80 and even *TRL*_20_ was correlated to *TRL*_60_ at *r* = 0.62, which was highly significant (*P* < 0.01) (Supplementary Table S1). Lower correlations were found with *FRL*. For example, *FRL*_20_ was not significantly correlated to *FRL*_60_, meaning that high densities of fine roots in the superficial layer could not predict the values at 60 cm, which were mainly dependent on the capacity of the primary roots to colonize the deep layers. To take into account whether or not the accession could maintain fine root colonization in the deep layers, a new derived trait was introduced: the coefficient of maintenance for fine roots (${\upalpha}_{FR{L}_{40\_60}}$) between 40 and 60 cm. A negative correlation was found between ${\upalpha}_{FR{L}_{40\_60}}$ and *FRL*_20_ (*r* = − 0.49, *P* < 0.01), meaning that strong colonization by fine roots at the surface was associated with a large decrease between 40 and 60 cm in depth. Likewise, *BI* displayed significant correlations between consecutive layers, but not between the superficial and deep layers. No significant correlation for *DIAM* _ *TR* between 20 and 40 cm was found, whilst it was highly significant between 40 and 60 cm (*r* = 0.86, *P* < 0.01). For this reason, a new derived trait was introduced: the reduction of diameter between 20 and 60 cm, $\kern0.5em {RED}_{T{R}_{20_{60}}}$. This trait was significantly and negatively correlated to *DIAM* _ *TR*_60_ (*r* = − 0.79, *P* < 0.01), meaning that the larger the diameter of primary roots at 60 cm depth, the lower was its reduction compared to the superficial layer (Supplementary Table S1).

### Determination of a Set of Root Architectural Traits

Considering a set of 28 root traits with some of them displaying a relatively large variation within and between each layer and based on a Pearson correlation analysis to identify the relationship between traits, a reduced subset of eight traits was extracted to characterize root architecture and development. These traits were:

- Fine root length at 20 cm (*FRL*_20_), quantifying the intensity of soil exploration in the superficial layer. It was highly correlated to *RL* at 20 cm (*r* = 0.99, *P* < 0.01) (Supplementary Table S1).
- Thick root length at 40 cm (*TRL*_40_), quantifying the level of colonization by the primary roots in the intermediate layer, a condition prior to the emergence of secondary and tertiary roots for intense exploration in the layer. It was also a good proxy of total root volume (and thus root biomass), because of its high correlation to *TOT*_*VOL*_ (*r* = 0.92, *P* < 0.01). It was also discriminant with the water treatments between the accessions (Supplementary Table S1).
- Thick root length at 60 cm (*TRL*_60_), measuring the ability of an accession to explore deeper layers, which is a key mechanism of adaptation to maintain water uptake during drought spells.
- Branching index at 40 cm (*BI*_40_), measured by the ratio between fine and thick root lengths (and not by the ratio of the number of fine roots generated per unit of thick root length). This is a fundamental structural trait, differing within accessions (Bañoc et al. 2000; Nibau et al. 2008; Gu et al. 2017). It manages the exploration intensity in a layer and the total explored soil volume (Gu et al. 2017). *BI*_40_ was the most representative *BI* value and was well correlated to *BI*_20_ and *BI*_60_ (*r* = 0.60, *P* < 0.05, *r* = 0.73, *P* < 0.01 respectively) (Supplementary Table S1).
- Maintenance coefficient of fine roots between 40 and 60 cm (${\upalpha}_{FR{L}_{40\_60}}$), measuring the ability of an accession to maintain intensive soil exploration below 40 cm. It was negatively correlated to *BD*_40_ and  *FRL*_20_, which was associated with the superficial root system.
- Thick root diameter at 60 cm (*DIAM* _ *TR*_60_), if the level of colonization at 60 cm was determined by *TRL*_60_, the accession’s ability to explore deeper layers, below 60 cm, can be approached by *DIAM* _ *TR*_60_.
- Thick Root diameter reduction between 20 and 60 cm (${RED}_{T{R}_{20_{60}}}$), as a predictor of the maximum potential depth.
- Total root volume (*TOT*_*VOL*_), representing the total investment of the accession in the root system and was considered as a proxy of total root biomass.

### Root Architecture Variability and Accession Clustering Under Irrigated Conditions

A Principal Component Analysis (PCA) was carried out on the above eight traits. *TOT*_*VOL*_ was used as a supplementary variable, considering the result of the combination of the other seven variables. The number of PCs to be kept was based on the Kaiser criterion, for which only components with eigenvalues ≥1 were chosen (Kaiser 1958). The first three PCs accounted for 90% of the total variation in root traits across the 17 rice accessions (Table 3), giving high consistency to the variable selection. The first two PCs explained 44.21% and 27.85% of the variance, respectively, both totalizing 72% and thus accounting for the main part of the variance in the original dataset. The third component (PC-3) accounted for 17.92% of the variance. The first principal component (PC-1) was characterized by *DIAM* _ *TR*_60_, *FRL*_20_ and *TRL*_40_, the second principal component (PC-2) accounted primarily for *BI*_40_, ${\upalpha}_{FR{L}_{20\_40}}$ and *TRL*_60_ (Table 3). The third principal component (PC-3) was characterized by $\kern0.50em {RED}_{T{R}_{20_{60}}}$, ${\upalpha}_{FR{L}_{20\_40}}$ and *TRL*_40_ (Table 3).

**Table 3:** Trait loading scores of the selected root traits and the proportion of variation for each principal component under irrigated conditions

Trait	Factor loadings		
	PC-1	PC-2	PC-3
*FRL*_20_	18.98	9.75	2.07
*TRL*_40_	18.57	4.78	19.10
*TRL*_60_	14.87	20.67	6.09
*BI*_40_	3.33	36.56	3.00
*DIAM* _ *TR*_60_	27.82	1.06	7.07
${\upalpha}_{FR{L}_{40\_60}}$	0.94	26.71	29.39
${RED}_{T{R}_{20_{60}}}$	15.49	0.48	33.26
Eigenvalue	3.09	1.95	1.25
Percentage of variance (%)	44.21	27.85	17.92
Cumulative percentage of variance (%)	44.21	72.07	89.98

**Fig. 2:**
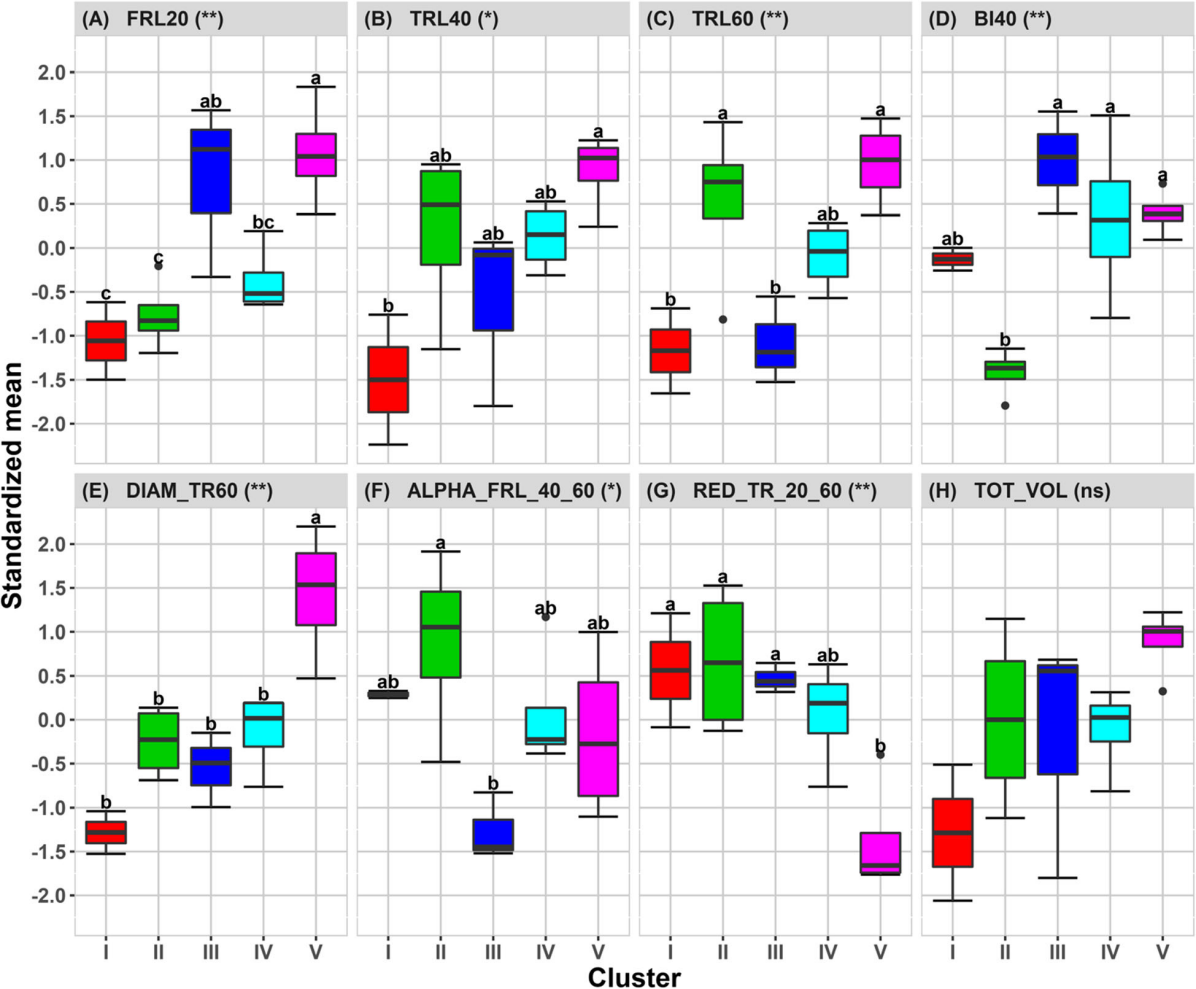
Box-plots of the standardized means of the eight selected root traits among the 17 accessions gathered in 5 clusters under irrigated conditions. The symbol in brackets indicates the cluster effect (** significant at 1%, * significant at 5% and ^*ns*^ *not significant*). *The averages followed by the same letter do not differ statistically according to the Tukey test* *(p-value 0.05)*

Variable representation and accession distribution in the PC-1 *vs* PC-2 planes are shown in Supplementary Fig. S3. The accessions were classed by HCA in five main clusters (I, II, III, IV and V) according to selected traits (Supplementary Fig. S4). The number of clusters was determined by the minimum number that explained the maximum of root trait variation. The pattern of distribution of the accessions within clusters appeared independent from their geographic origin (Supplementary Figs. S1 and S4). Multiple comparisons of means between clusters across the traits at 95% confidence level (Tukey’s Honest [HSD] test) are presented by letters (Fig. 2). The cluster effect was significant for all traits, except for *TOT*_*VOL*_.

A schematic representation of the overall cluster traits is given in Supplementary Fig. S5. Cluster I contained the accessions (Ma Hae and Lambayque 1) that had a globally “weak” root system: primary roots were thin (low *DIAM* _ *TR*_60_ and high $\kern0.5em {RED}_{T{R}_{20_{60}}}$) and ensured poor colonization in the medium and deeper layers (low *TRL*_40_ and  *TRL*_60_). Although there were no significant differences in total root volume (*TOT*_*VOL*_), it was found that the accessions in cluster I had the lowest values for *TOT*_*VOL*_ within all the clusters, which could be explained by the low values for thick roots (*TRL*_40_ and *TRL*_60_) (Fig. 2). These accessions did not develop a strong net of fine roots so far down, or even in the superficial layer, where *FRL*_20_ had the lowest value between clusters (Fig. 2 and Supplemetary Fig S5a).

Conversely, cluster V contained the accessions (IAC 25, HD 1–4, Guarani and Cirad 409) with a “strong” root system, setting it apart through its characteristics from all the other clusters (Fig. 2): highest biomass (estimated by total root volume), thick primary roots (high  *DIAM* _ *TR*_60_) with a slight decrease in diameter along the profile (small $\kern0.5em {RED}_{T{R}_{20_{60}}}$), ensuring good colonization of the medium and deep layers (high *TRL*_40_ and *TRL*_60_) (Supplementary Fig. S5e). As *TRL*_40_ and *TRL*_60_ were at the same levels, it also indicated a large number of primary roots crossing the whole 20–40 cm layer. Consequently, fine roots were well developed in the superficial layer but poorly maintained between 40 and 60 cm.

The main characteristics of the 4 accessions found in cluster II (Peek, Indane, Early Mutant IAC 165 and Cha Phu Ma) was a low branching index (*BI*_40_) and a consecutive poor length density for fine roots at the surface (low *FRL*_20_), but good conservation between 40 and 60 cm (high ${\upalpha}_{FR{L}_{40\_60}}$) (Fig. 2). Primary roots had a low-medium diameter (*DIAM* _ *TR*_60_), ensuring, by their numbers, good colonization of the medium and deep layers (high *TRL*_40_ and *TRL*_60_) (Supplementary Fig. S5b). Combined with the large decrease in diameter between 20 and 60 cm, the potential ability to explore deep layers was low.

Cluster III contained 3 accessions (IAC 164, Douradão and Cirad 392) that had a typical superficial root system: thin primary roots (low-medium *DIAM* _ *TR*_60_), with poor colonization at depth (mean *TRL*_40_ and low *TRL*_60_), a highly branched system (*BI*_40_) inducing a high density of fine roots in the superficial layer (*FRL*_20_) (Figs. 2 and Supplementary Fig. S5c). Lastly, cluster IV contained 4 accessions (Soberana, Ghaselu Map, Gemjya Jyanam and Dakpa) that had mean values for all the traits, illustrated by its position in the center of PCA 1–2 (Supplementary Fig. S3). Moderate values were found for thick root length (*TRL*_40_ and  *TRL*_60_) and, by contrast with cluster II, the accessions contained in cluster IV were more branched (high *BI*_40_) (Fig. 2 and Supplementary Fig S5d).

### Stability of Root Trait Correlations Under Two Water Regimes

As already seen, the studied traits had some correlations between them under a non-limiting water regime (Supplementary Table S1). With the response of the accessions to drought, the question was to know whether or not the correlations persisted, and what the functional meanings of hypothetical changes were. A Pearson correlation coefficient analysis revealed medium to strong correlations between measured (*FRL*_20_, *TRL*_40_ and *TRL*_60_) and derived root traits (*BI*_40_, *DIAM* _ *TR*_60_, ${\upalpha}_{FR{L}_{40\_60}}$, ${RED}_{T{R}_{20_{60}}}$ and *TOT*_*VOL*_) under full irrigation, which remained unchanged across both water treatments (Table 4). Thus, whatever the water regime, the total root volume (*TOT*_*VOL*_) used as a proxy of total root biomass was highly correlated to primary root lengths (*TRL*_40_ and *TRL*_60_), which are necessary conditions for the development of fine roots in the superficial layer (*FRL*_20_). For necessary but not sufficient conditions, *FRL*_20_ was not linked to *TRL*_60_ in any water treatment. Another main determinant of the total root volume, the thickness of primary roots (*DIAM* _ *TR*_60_), was also highly correlated to *TOT*_*VOL*_ in all the situations, while the branching index (*BI*_40_) had no significant effect. In both the irrigated and drought treatments, ${RED}_{T{R}_{20_{60}}}$ was highly and negatively correlated to the primary root diameter (*DIAM* _ *TR*_60_): the thinner the primary root was, the larger was its diameter reduction at a depth of 60 cm, giving consistency to using a combination of both traits to predict potential maximum depth.

**Table 4:** Phenotypic correlations for the 8 selected root traits among the 17 rice accessions under two water conditions

Trait	*FRL*_20_	*TRL*_40_	*TRL*_60_	*BI*_40_	*DIAM* _ *TR*_60_	${\upalpha}_{FR{L}_{40\_60}}$	${RED}_{T{R}_{20_{60}}}$	*TOT*_*VOL*_
*FRL*_20_	–	0.044^ns^	− 0.095^ns^	0.361^ns^	0.186^ns^	− 0.371^ns^	0.293^ns^	**0.534**^**a**^
*TRL*_40_	0.452^ns^	–	**0.828**^**b**^	**−0.585**^**b**^	**0.775**^**b**^	−0.142^ns^	**− 0.536**^**a**^	**0.761**^**b**^
*TRL*_60_	0.267^ns^	**0.801**^**b**^	–	**−0.597**^**b**^	**0.852**^**b**^	0.236^ns^	**− 0.712**^**b**^	**0.730**^**b**^
*BI*_40_	**0.485**^**a**^	−0.057^ns^	− 0.339^ns^	–	− 0.347^ns^	− 0.229^ns^	0.217^ns^	− 0.217^ns^
*DIAM* _ *TR*_60_	**0.580**^**a**^	**0.601**^**a**^	**0.642**^**b**^	0.259^ns^	–	0.113^ns^	**− 0.742**^**b**^	**0.760**^**b**^
${\upalpha}_{FR{L}_{40\_60}}$	**−0.489**^**a**^	−0.192^ns^	0.163^ns^	**− 0.499**^**a**^	0.130^ns^	–	− 0.380^ns^	− 0.171^ns^
${RED}_{T{R}_{20_{60}}}$	− 0.440^ns^	− 0.183^ns^	− 0.218^ns^	− 0.358^ns^	**− 0.791**^**b**^	−0.131^ns^	–	− 0.364^ns^
*TOT*_*VOL*_	**0.625**^**b**^	**0.923**^**b**^	**0.715**^**b**^	0.009^ns^	**0.528**^**a**^	−0.298^ns^	− 0.118^ns^	–

^ns^Non-significant; ^a^ and ^b^ significant by the t-test at 5 and 1% probability, respectively. Values for irrigated conditions are below the diagonal and for drought above the diagonal

The main changes found between irrigated and drought conditions were related to the branching index (*BI*). Under irrigated conditions, *BI*_40_ was positively correlated to fine root length in the upper layer (*FRL*_20_, *r* = 0.485, *P* < 0.05), but not under drought (Table 4). Also under irrigated treatment, it was negatively correlated to ${\upalpha}_{FR{L}_{20\_40}}$ (*r* = − 0.499, *P* < 0.05), but not under drought. On the other hand under drought, *BI*_40_ was highly and negatively correlated to thick root length (*TRL*_40_ and *TRL*_60_), whereas it was not so under irrigated conditions: under drought, a high branching index was detrimental to deep colonization by primary roots, leading to a decrease in secondary root development at 40–60 cm and a lowering of *BI* and ${\upalpha}_{FR{L}_{20\_40}}$ in those layers.

### Genotype Root Response to Drought

With the application of water deficit after panicle initiation and up to heading, the root systems exhibited different growth responses. In comparison with the fully irrigated treatment, accessions under drought exhibited: 1) a reduction in overall biomass accumulation (as estimated by the total root volume at the time of heading) in relation to control plants; 2) its stability, or even 3) an increase (Supplementary Fig. S6). In the first case, root growth was slowed down by drought in a set of nine accessions (Ma Hae, Douradão, Guarani, HD 1–4, Cha Phu Ma, Gemjya Jyanam, IAC 164, Early Mutant IAC 165 and Soberana). This occurred with different amplitudes, Ma Hae and Douradão being little affected (reduction of 11.9% and 13.8%, respectively) while IAC 164, Early Mutant IAC 165 and Soberana displayed a larger reduction (26.7%, 26.9% and 29.5%, respectively) (Table 5). It should be noted that these accessions are genetically close to each other and in particular, IAC 164 and Early Mutant IAC 165 are derived from the same cross (Silva et al. 1999). The cumulative root profile also provided information on the variation patterns: the decrease when compared to the control plants was distributed throughout the profile (Guarani) (Supplementary Fig. S6k), or only in the superficial layers (HD 1–4, Cha Phu Ma, Gemjya Jyanam or IAC 164) (Supplementary Figs. S6l-o).

**Table 5:** Relative variation of the selected root traits from irrigated to water deficit conditions

Accessions	RG	Drought effect (VAR*∆*_DC/C_)															
		*TOT*_*VOL*_		*FRL*_20_		*TRL*_40_		*TRL*_60_		*BI*_40_		*DIAM*_*TR*60_		${\alpha}_{FR{L}_{40\_60}}$		${RED}_{T{R}_{20_{60}}}$	
Lambayque 1	I	0.596	a	1.492	a	0.890	ab	1.405	a	-0.090	a	0.071	a	0.193	abc	-0.571	b
Cirad 392	I	0.559	a	0.436	abcd	1.201	a	1.132	ab	-0.193	a	0.047	ab	0.221	abc	-0.852	b
Peek	I	0.472	ab	0.959	abc	0.173	abc	0.725	ab	0.693	a	0.026	abc	-0.156	bc	-0.500	b
Cirad 409	I	0.341	abc	0.083	bcd	0.228	abc	0.207	ab	-0.168	a	0.008	abc	0.013	abc	-1.426	b
Indane	I	0.214	abcd	0.948	abcd	0.033	abc	0.087	ab	0.602	a	-0.004	abc	-0.114	bc	-0.257	b
IAC 25	II	0.071	bcde	0.269	abcd	-0.037	bc	0.009	ab	-0.197	a	0.019	abc	0.350	abc	0.150	b
Ghaselu Map	II	0.005	bcde	1.254	ab	-0.452	c	-0.570	b	0.823	a	-0.031	bc	-0.252	c	1.698	b
Dakpa	II	0.001	bcde	1.207	ab	-0.531	c	-0.333	ab	1.454	a	-0.015	abc	0.342	abc	0.092	b
Ma Hae	III	-0.119	cde	-0.339	d	-0.180	bc	0.033	ab	0.033	a	0.019	abc	0.331	abc	-0.483	b
Douradão	III	-0.138	de	0.004	bcd	-0.396	c	0.263	ab	0.977	a	0.037	ab	0.273	abc	-0.211	b
Guarani	III	-0.160	de	0.138	bcd	-0.362	c	-0.506	b	0.547	a	-0.061	c	-0.449	c	13.708	a
HD 1-4	III	-0.162	de	0.012	bcd	-0.500	c	-0.265	ab	0.504	a	-0.016	abc	0.122	abc	3.166	b
Cha Phu Ma	III	-0.181	de	-0.123	cd	-0.138	bc	-0.012	ab	1.007	a	0.015	abc	-0.123	bc	-0.765	b
Gemjya Jyanam	III	-0.185	de	-0.113	cd	-0.466	c	-0.044	ab	0.552	a	0.004	abc	0.286	abc	-1.169	b
IAC 164	III	-0.267	e	-0.310	cd	-0.574	c	-0.111	ab	0.029	a	-0.016	abc	0.952	a	0.083	b
Early Mutant IAC 165	III	-0.269	e	-0.122	cd	-0.369	c	-0.318	ab	0.446	a	-0.007	abc	-0.029	abc	-0.263	b
Soberana	III	-0.295	e	-0.145	cd	-0.526	c	-0.202	ab	0.296	a	0.007	abc	0.821	ab	-0.205	b
Accession effect		**		**		**		**		ns		**		**		**	

RG: response group. The averages followed by the same letter do not differ statistically according to the Tukey test (*p-value* 0.05). One-way ANOVA results are presented with respective significances for the accession. ^ns^ Non-significant; * and ** significant by the t-test at 5 and 1% probability, respectively

In three accessions (IAC 25, Ghaselu Map and Dakpa) (Supplementary Figs. S6f-h), overall growth was maintained under drought, with different patterns: unchanged profile in IAC 25, or the reduced colonization of deep and intermediate layers, catching up in the superficial layer with the other two materials. Lastly, five accessions had a net increase in total root volume under drought (Lambayque 1, Cirad 392, Peek, Cirad 409 and Indane) (Supplementary Figs. S6a-e), well distributed between all the layers in Lambayque 1, Cirad 409 and Indane, more in the superficial layers for Cirad 392 and in the deeper ones for Peek.

**Fig. 3:**
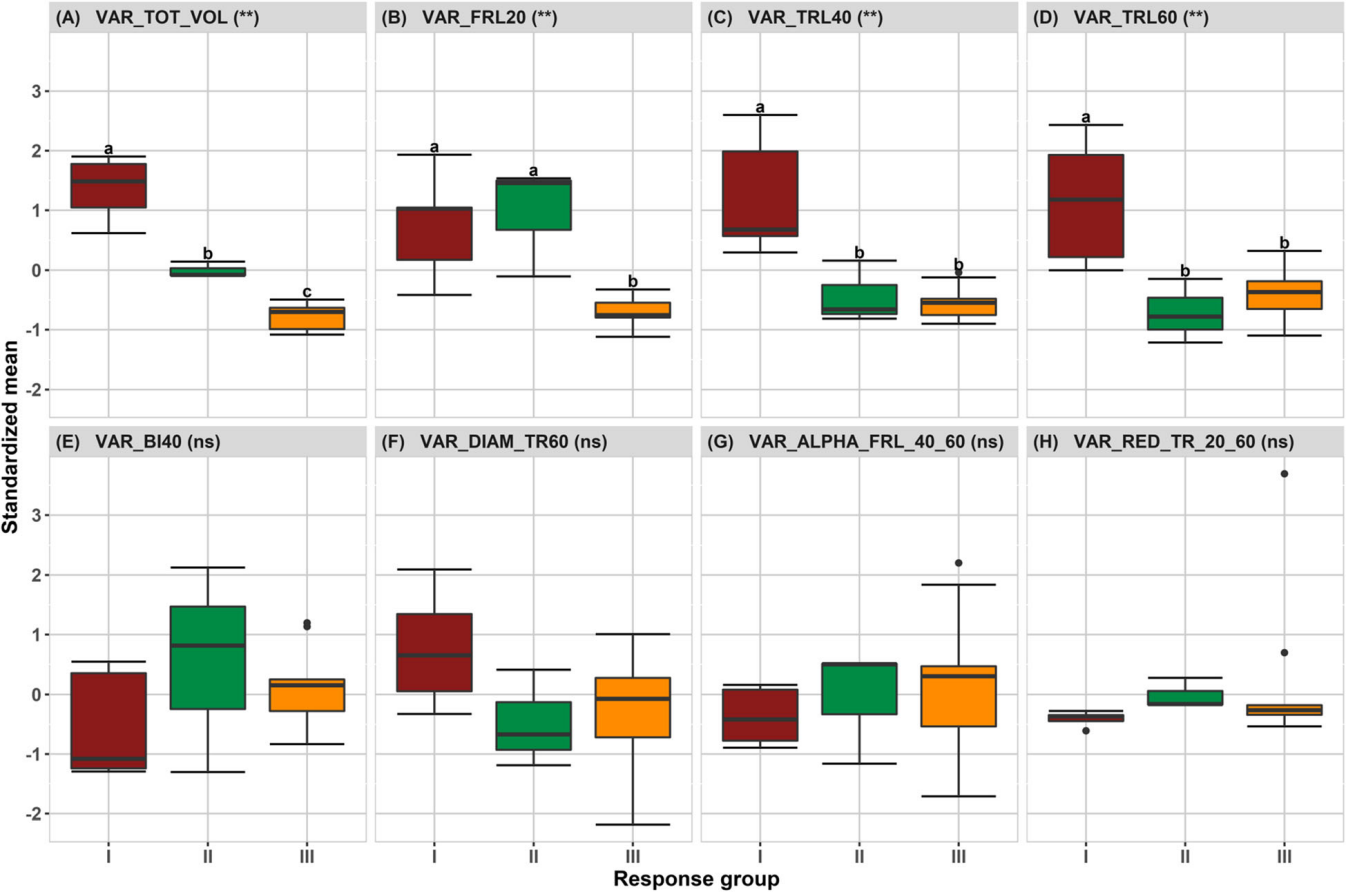
Box-plots of the standardized relative variations of the eight selected root traits among the 17 accessions gathered in 3 response groups (RG). The symbol in brackets indicates the response group effect (** significant at 1% and ^*ns*^ *not significant). The averages followed by the same letter do not differ statistically according to the Tukey test (p-value 0.05). I, II, III refer to response groups RG I, RG II and RG III*

The accessions could be split into three response groups (RG) to drought, based on their ability to modify assimilate allocation to roots under contrasting water regimes: decrease, stability or increase. When investigating the relative variations between irrigated and drought conditions (*VAR∆*_*DC*/*C*_), significant differences were found between the accessions for all traits, except for branching index (*BI*_40_) (Table 5). We then studied the response group effect on the eight trait variations (Fig. 3): significant differences were found for $\kern0.75em {\Delta }_{TOT_{VOL}}$, *∆*_*FRL*20_,  *∆*_*TRL*40_ and *∆*_*TRL*60_. Consistently, *∆*_*BI*40 _ had a non-significant response group effect, in relation to its high variability. ${\Delta _{\alpha}}_{FR{L}_{40\_60}\kern0.5em }$ and $\kern0.5em {\Delta}_{RED_{T{R}_{20_{60}}}}$ had little and non-significant variations.

Clustering highlighted the role of thick root changes in total root volume variation: response group I (RG I) contained five accessions (Lambayque 1, Cirad 392, Peek, Cirad 409 and Indane) that increased total root volume under drought, displaying a significant increase in thick root length at 40 and 60 cm in relation to the irrigated treatment, as well as an increase in thick root diameter at 60 cm (though not significant), while all these traits decreased in the response groups II and III (RG II and RG III). The accessions in RG I were able to colonize deeper layers better under drought than those in RG II and RG III. Interestingly, the accessions in RG II, which contained three accessions (IAC 25, Ghaselu Map and Dakpa), displayed a decrease in primary root length in the deeper layers (40 and 60 cm) in the drought treatment, while there was increasing fine root colonization in the superficial layer (Fig. 3).

**Fig. 4:**
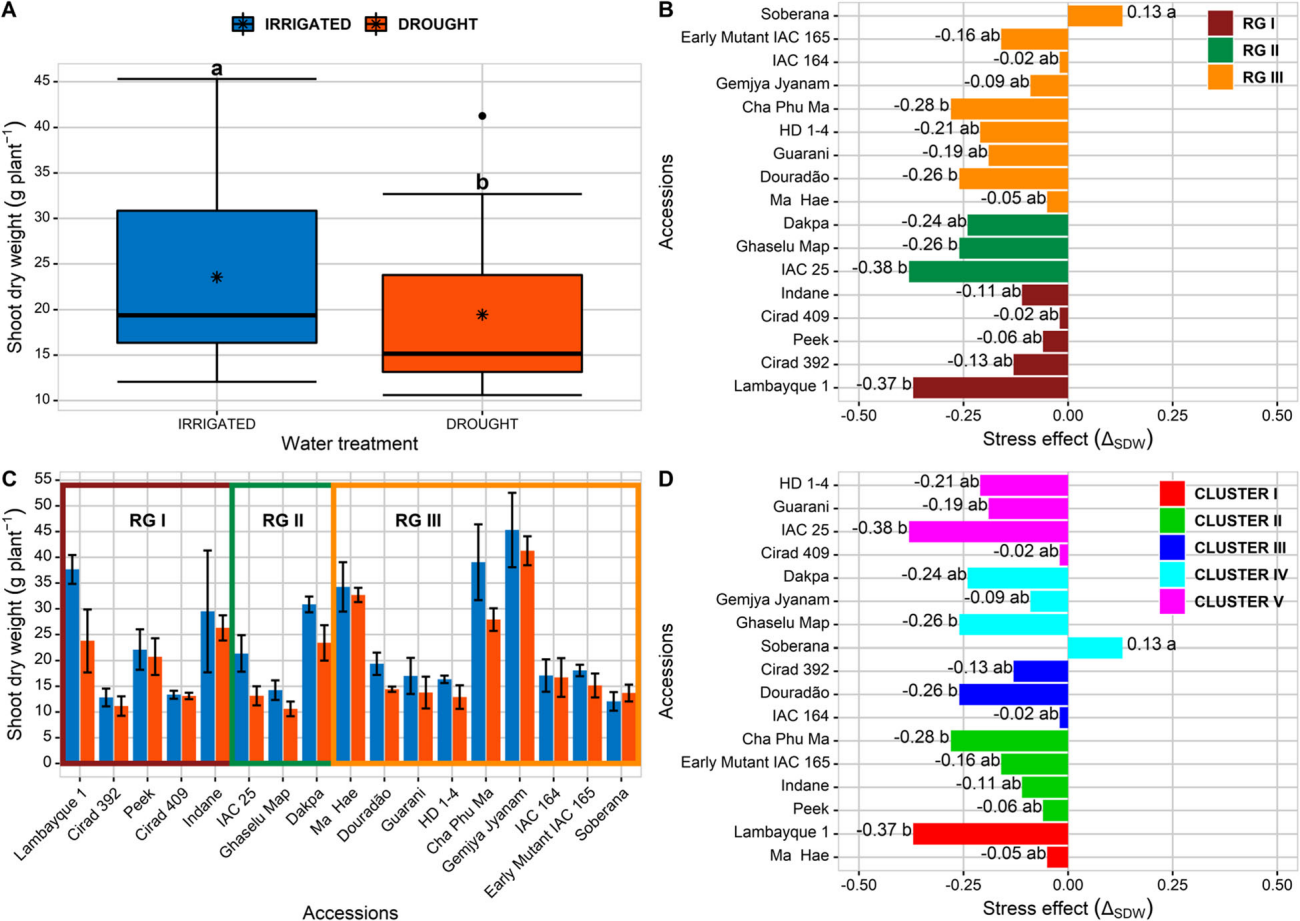
Shoot dry weight (*SDW*) of 17 accessions under two water conditions. (A) *Box-plots showing the SDW under irrigated and drought conditions*, (B) *Shoot drought response *(*∆*_*SDW*_) *for each accession according to its membership to one response group*, (C) *Shoot dry weight of each accession under two water conditions*, (D) *Shoot drought response* (*∆*_*SDW*_) *for each accession according to its membership to one cluster*. * *The averages followed by the same letter do not differ statistically according to the Tukey test (p-value 0.05)*. *RG: response group. Values are means of three replicates ± SD*

The shoot growth was significantly affected by water deficit and the global mean decreases from 23.9 g/plant to 19.5 g/plant in irrigated and drought treatments respectively (Fig. 4a). The large differences in shoot dry weight between accessions (Fig. 4c) were mainly explained by differences in earliness in our panel, the vegetative phase extending from 24 days (Cirad 409) to 42 days (Cha Phu Ma). In all accessions a decrease in shoot dry weight with water deficit was observed, except in Soberana (Fig. 4c). The reduction of shoot dry weight with drought ranged from 37% to 38% in Lambayque and IAC 25 to 2% in Cirad 409 and IAC 164, while Soberana had an increase of 13% (Fig. 4b). Due to high variability, differences were significant only between Soberana in one hand and IAC 25, Lambayque, Cha Phu Ma, Douradão and Ghaselu Map on the other hand. Interestingly, it was observed an uncoupling between the membership of an accession to a root response group and its shoot response (Fig. 4b): all the three groups gathered accessions with low or high reduction in shoot growth. This means that the accelerated root biomass accumulation (*TOT*_*VOL*_) under drought observed in accessions from RG I was not systematically associated with the maintenance of shoot growth. The same observation was done with the clustering achieved on structural traits (Fig. 4d): whatever the type of root system in irrigated conditions, no correlation was found with the shoot growth capacity under water deficit.

## Discussion

The SITIS phenotyping platform provided quality data for assessing the root system throughout plant development up to heading, from the top layer and down to 60 cm, in 20 cm layers. Despite high CVs, as commonly found in root studies (Courtois et al. 2013; Terra et al. 2016; Li et al. 2017), the SITIS platform brought out systematic highly significant effects of accessions and water treatments, as well as frequent interaction effects (Table 2), confirming its good performance for root screening.

In trials in controlled environments, the use of non-invasive techniques, such as magnetic resonance imaging (Schulz et al. 2013; van Dusschoten et al. 2016; Pflugfelder et al. 2017) and rhizotrons (Jeudy et al. 2016; Gioia et al. 2017; Jia et al. 2019; Bettembourg et al. 2017), is not new and has been practised in high-throughput phenotyping of root systems. Here, by running WinRhizo software on scanned images, the study attempted to analyze root traits beyond direct commonly analyzed traits, such as root length (Kondo et al. 2000; Samejima et al. 2005; Qu et al. 2008; Zhan et al. 2015; Phung et al. 2016) and root length density (Yang et al. 2004; Zhang et al. 2009; Ganapathy et al. 2010; Courtois et al. 2013). Some traits may appear more useful than others for characterizing root systems, depending on the objective of the study. For instance, Fageria (2013) stated that root length was the best parameter regarding water and nutrient uptake, while others (Singh et al. 2013; Hazman and Brown 2018; Solis et al. 2018) insisted on the maximum root depth in relation to drought tolerance. Here, dealing with the response of root systems to drought, we attempted to reconstruct root structures and development patterns, which are poorly reported in the literature but used in complex models as described by Tonglin et al. (2006); Fang et al. (2009); Pagès and Picon-Cochard (2014).

The characterization (albeit simplified) of root architecture can help in assessing the explored volume and its intensity of exploitation. Thus, using WinRhizo software, we separated roots into: 1) fine roots (*FRL*) with a diameter less than 0.5 mm, which was representative of secondary and tertiary roots; and 2) thick roots (*TRL*) with a diameter greater than 1 mm, which ensured taking into account only primary roots (Price et al. 1989; Bauhus and Messier 1999; Dien et al. 2017). This simple procedure allowed us to analyze the ability of soil exploration (laterally and vertically) by primary and secondary/tertiary roots. Fine roots are associated with water uptake and nutrient uptake (in particular nutrients with low mobility such as phosphorus) (Blouin et al. 2007; Henry et al. 2011; Comas et al. 2013; Gu et al. 2017) and thick roots (larger root diameter) are associated with deep volume exploration and greater soil penetration ability, mainly through hardpans under drought (Yu et al. 1995; Clark et al. 2008; Bengough et al. 2011; Lynch 2014). In addition, considering direct variables such as *DIAM* _ *TR*_60_ and new architectural and spatial variables such as *BI*_40_, ${\upalpha}_{FR{L}_{40\_60}}$ and ${RED}_{T{R}_{20_{60}}}$ and through Pearson’s correlation (Supplementary Table S1) between 28 root traits, we were able to identify eight relevant traits to characterize root structure (Table 3).

By analyzing the 17 rice accessions using a PCA with the eight selected traits, it was possible to explain 90% of total variability on the main three axes, and 72% with the first two axes (Supplementary Fig. S3). This value was close to the values found by Courtois et al. (2013) [74.5%] and Phung et al. (2016) [69.6%], whose genetic panel included japonica and indica accessions. The present study focused on a sole japonica set and separated new variables such as  *FRL*, *TRL* and *DIAM* _ *TR* within each layer. By PCA analysis, we succeeded in identifying some root system types differing in growth patterns and spatial colonization (Fig. 2 and Supplementary Fig. S5). The values found (Supplementary Fig. S7), were in a similar range to those found by Dien et al. (2017); Gu et al. (2017) and Li et al. (2017).

The combination of the eight variables generated five main clusters, significantly separated by HCA analysis. The clusters obtained under irrigated conditions firstly separated accessions into overall “strong” and “weak” root systems, according to the total volume, or “superficial” and “deep” systems, depending on the distribution of thick and fine roots throughout the soil profile. Interestingly, accessions from cluster V (Fig. 2 and Supplementary Figs. S3 and S5E) combined a high density of fine roots in the superficial layer and abundant thick roots in the deeper layers. It is well known that the tropical japonica subspecies has thick, deep and little branched roots, associated with a high root penetration index (Babu et al. 2001). In comparison, the indica subspecies has a more superficial and branched root system (Lafitte et al. 2001). It is also known that the japonica subspecies displays wide variation in root anatomical traits (stele diameter and xylem structure), whereas the indica subspecies has wide variation in root architecture (root thickness and branching) (Kondo et al. 2003; Uga et al. 2009; Henry et al. 2011). Here, we found a wide diversity of root architectures in a reduced panel of japonica accessions. Beyond this simplified classification, the radar chart gives a visualization of the different root types in all their dimensions, with possible applications in genetic improvement (Supplementary Fig. S5). As noted by Kondo et al. (2003); Lynch and Brown (2012); Trachsel et al. (2013) and Lynch (2013), the root system pattern found in cluster I, highly branched with high fine root density in the surface layer is known to be more appropriate to low fertility soils (i.e. when phosphorus or potassium are limiting), and under non limiting water conditions. It is also more adapted to quickly capture water in recovering process after a dry spell rather than to tolerate long drought period. Oppositely, the root system pattern found in cluster V is suitable in soils with a high risk of nutrient leaching and low water retention capacity, in which water and solubilized nutrients easily migrate from the topsoil and can be found in deeper layers (Trachsel et al. 2013). Furthermore, cluster V root type would be favorable for plant to endure drought periods, by its ability to uptake water stored in deeper layers (Mambani and Lal 1983; Gowda et al. 2011; Wasson et al. 2012; Trachsel et al. 2013).

Under water-limited conditions, the response of the root system is known to be plastic (Ahmadi et al. 2014; Kameoka et al. 2016; Khan et al. 2016; Sandhu et al. 2016), which was confirmed in this study. Here, for some accessions, we found a net increase in total root volume (used as a proxy of root biomass) in response to drought, as already found by Ding et al. (2015) and Li et al. (2017). Originally, the study showed that the root response to drought was not dependent on its classification under irrigated conditions. Two accessions from the same cluster responded differentially to water deficit: for example in cluster V containing accessions with a “strong” root system under irrigated conditions (Supplementary Figs. S3 and S5), the accession HD 1–4 displays a large reduction in its root growth (Table 5), while Cirad 409 increases it. Oppositely, Lambayque 1 developed a poor root system under irrigated conditions (cluster I), but was able to stimulate its root system in response to drought (RG I) (Supplementary Fig. S3, Fig. 2 and Table 5). We can therefore consider that inductive traits, triggered in response to drought, are independent from the constitutive traits expressed under non-limiting water conditions. Thus, the results found here could be used to identify the genetic basis of specific root traits, and help in characterizing traits suitable for targeted selection and breeding of new rice cultivars for efficient use of water and nutrients.

The shoot response to drought, in relation with root response, deserves also some attention. Globally, under a moderated drought as applied in our experiment, shoot was more affected than root growth, inducing a well-known increase in root/shoot ratio (Kondo et al. 2000; Price et al. 2002b; Gowda et al. 2011; Xu et al. 2015). In these conditions, some accessions presented a stimulated root biomass accumulation (*TOT*_*VOL*_) under drought in comparison with control plants (accessions from RG I), that did not confer any advantage in the maintenance of shoot growth. More, the study showed also an uncoupling between the structural traits of the root system in irrigated conditions (defined by the 5 clusters) (Supplementary Fig. S5) and the shoot response under drought (Fig. 4 and Table 5). The present study did not have the means to decipher this disconnection between structural/morphological traits and physiological responses to water deficit. It is known that under progressive drought, shoot expansion is affected before any effect on leaf photosynthesis (Boyer 1970; Muller et al. 2011). With the reduced shoot demand, assimilates are derived to root system, for growth and storage, which explains in turn the root/shoot increase (Lemoine et al. 2013; Xu et al. 2015; Zhang et al. 2016). The rules of assimilate partitioning are also depending on the shoot water status, with a critical threshold for expansion process that can impact the carbon flow (Wilson 1988; Lemoine et al. 2013). The response to drought seems genetically determined, each accession having its own cursor placement in allocating preferably assimilates for shoot expansion or root growth or storage, independently of the initial root system structural traits. This could be illustrated by the proximity of response of IAC 164, Early Mutant IAC 165 and Soberana, which are genetically close (Silva et al. 1999; Cargnin et al. 2008). Finally, we must be careful with the reliability of shoot data, because *SDW* was measured on one single plant per pipe while root volume was observed on the three plants. Despite any precaution in interpretation, the results found here are consistent with those found by Dien et al. (2017).

## Conclusions

From a methodological point of view, this study brought out some new structural traits for analyzing root system growth and development that can be collected without increasing the experimental work, merely by extracting more information from the scanned images and WinRhizo software analysis, and from the trait distribution within the three layers studied. The SITIS platform used for the experiment demonstrated its efficiency and ability to cope with more samples, thereby opening up the way to high-throughput phenotyping. In a recent experiment (data to be exploited), 184 accessions of the *PRAY* japonica panel, including our 20 accessions, were phenotyped and will be used in a GWAS perspective, comparing them for their root lengths and volumes, to which our new structural traits can be easily added.

This study demonstrated the wide diversity of root architectures existing in a reduced panel of tropical japonica rice accessions, which can be used in breeding programs in addition to the traits already evaluated. Moreover, it was found here that the root development pattern of an accession under non limiting water supply did not predict its adaptive or unadaptive response to drought, which needs to be considered in breeding programs.

## Additional files

Additional file 1: **Supplementary Fig. S1.** Geographical origin of the 20 tropical japonica rice accessions from the tropical japonica panel evaluated at SITIS.

Additional file 2: **Supplementary Fig. S2.** Meteorological conditions over experimental time. (DOCX 210 kb)

Additional file 3: **Supplementary Fig. S3.** Principal Component Analysis representation (plan 1-2) on the eight selected root traits among 17 rice accessions grown under irrigated conditions.

Additional file 4: **Supplementary Fig. S4.** Hierarchical Classification Analysis on the eight selected root traits among 17 rice accessions grown under irrigated conditions. *The 17 rice accessions were assigned to one of the five general clusters (Cluster I, Cluster II, Cluster III, Cluster IV and Cluster V). The eight root traits were the same as those used for the PCA in Table *3.

Additional file 5: **Supplementary Fig. S5.** Radar chart for the different root system profiles under irrigated conditions. *Each circle represents 20, 40, 60, 80 and 100% of variability found for each variable by the hierarchical cluster analysis (HCA).*

Additional file 6: **Supplementary Fig. S6** Cumulative root volume of the 17 accessions. *Root volume was measured by 4 cm layers from 60 cm depth to soil surface.*

Additional file 7:**Supplementary Fig. S7** Bar plots and standard error of the eight selected root traits for each accession under two water conditions. *Fine root length (FRL*_20_*), thick root length (TRL*_40_ *and TRL*_60_*), branching index (BI*_40_*), thick root diameter (DIAM* _ *TR*_60_*), coefficient of maintenance for fine roots between 40 and 60 cm (*${\alpha}_{FR{L}_{40\_60}}$*), thick root diameter reduction between 20 and 60 cm (*${RED}_{T{R}_{20_{60}}}$*) and total root volume (TOT*_*VOL*_*). Values are means of three replicates ± SD.*

Additional file 8: **Supplementary Table S1.** Pearson’s correlation matrix for 29 traits (28 root traits, and one shoot trait) among 17 rice accessions grown in a phenotyping platform across three layers under irrigated conditions.

## Data Availability

All data generated or analysed during this study are included in this published article. The phenotypic data are included in Supplementary Fig. S7.

## Abbreviations

NUE: Nutrient use efficiency
WUE: Water use efficiency
*QTLs*: Quantitative trait loci
SITIS: Integrated System for Drought-Induced Treatment
NPK: Nitrogen, phosphorus and potassium
FC: Field capacity
*WP*: Wilting point
*FTSW*: Fraction of transpirable soil water
*PI*: Panicle initiation
*T*_*max*_: Maximum temperature
*RL*: Root length
*RV*: Root volume
*RD*: Root diameter
*TOT*_*VOL*_: Total root volume
*TOT*_*RL*_: Total root length
${\upalpha}_{FR{L}_n}$: Coefficient of maintenance for fine roots
${\upalpha}_{TR{L}_n}$: Coefficient of maintenance for thick roots
*BI*: Branching index
*DIAM* _ *TR*_*n*_: Thick root diameter
${RED}_{T{R}_{20_{60}}}$: Thick root diameter reduction
ANOVA: Analysis of variance
PCA: Principal component analysis
HCA: Hierarchical cluster analysis
RG: Response group
HSD: Tukey’s Honest test
*VAR∆*_*DC*/*C*_: Response to drought
*FRL*: Fine root length
*TRL*: Thick root length
*SDW*: Shoot dry weight
