# Supplementary material for: Phenotyping Root Systems in a Set of Japonica Rice Accessions: Can Structural Traits Predict the Response to Drought?
Source: Rice (N Y). 2020 Sep 15;13:67. doi: 10.1186/s12284-020-00404-5 (PMC7492358; doi:10.1186/s12284-020-00404-5)
Supplement: Supplementary file 1 — Supplementary Fig. S1. Geographical origin of the 20 tropical japonica rice accessions from the tropical japonica panel evaluated at SITIS. [file 12284_2020_404_MOESM1_ESM.docx]

**
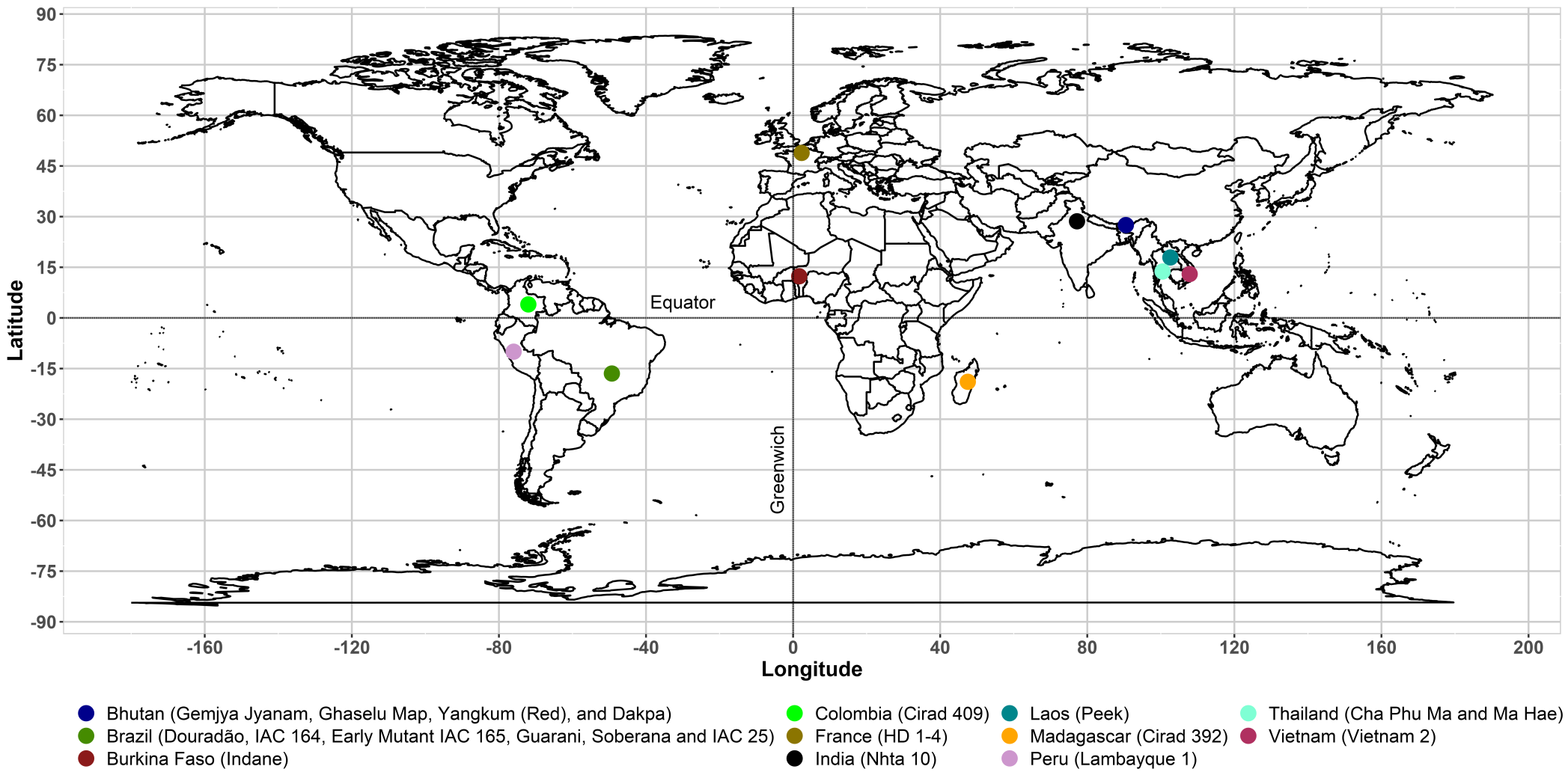
**

**Supplementary Fig. S1** Geographical origin of the 20 tropical japonica rice accessions from the tropical japonica panel evaluated at SITIS
