# Supplementary figures and images for: Phenotyping Root Systems in a Set of Japonica Rice Accessions: Can Structural Traits Predict the Response to Drought?
Source: Rice (N Y). 2020 Sep 15;13:67. doi: 10.1186/s12284-020-00404-5 (PMC7492358; doi:10.1186/s12284-020-00404-5)

**
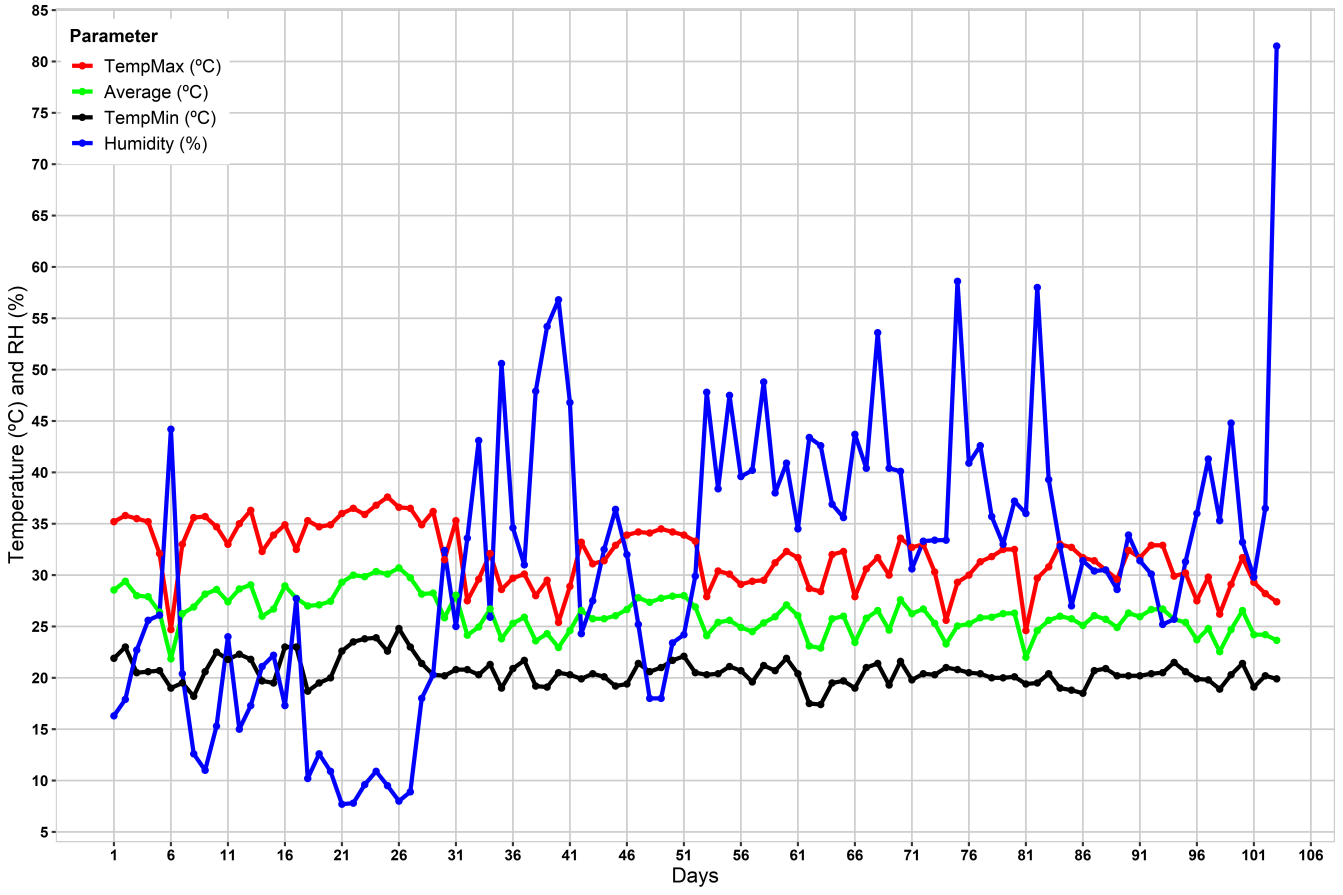
**

**Supplementary Fig. S2** Meteorological conditions over experimental time

Supplement: Supplementary file 2 — Supplementary Fig. S2. Meteorological conditions over experimental time. (DOCX 210 kb) [file 12284_2020_404_MOESM2_ESM.docx]
