# Supplementary material for: Phenotyping Root Systems in a Set of Japonica Rice Accessions: Can Structural Traits Predict the Response to Drought?
Source: Rice (N Y). 2020 Sep 15;13:67. doi: 10.1186/s12284-020-00404-5 (PMC7492358; doi:10.1186/s12284-020-00404-5)
Supplement: Supplementary file 3 — Supplementary Fig. S3. Principal Component Analysis representation (plan 1-2) on the eight selected root traits among 17 rice accessions grown under irrigated conditions. [file 12284_2020_404_MOESM3_ESM.docx]

**
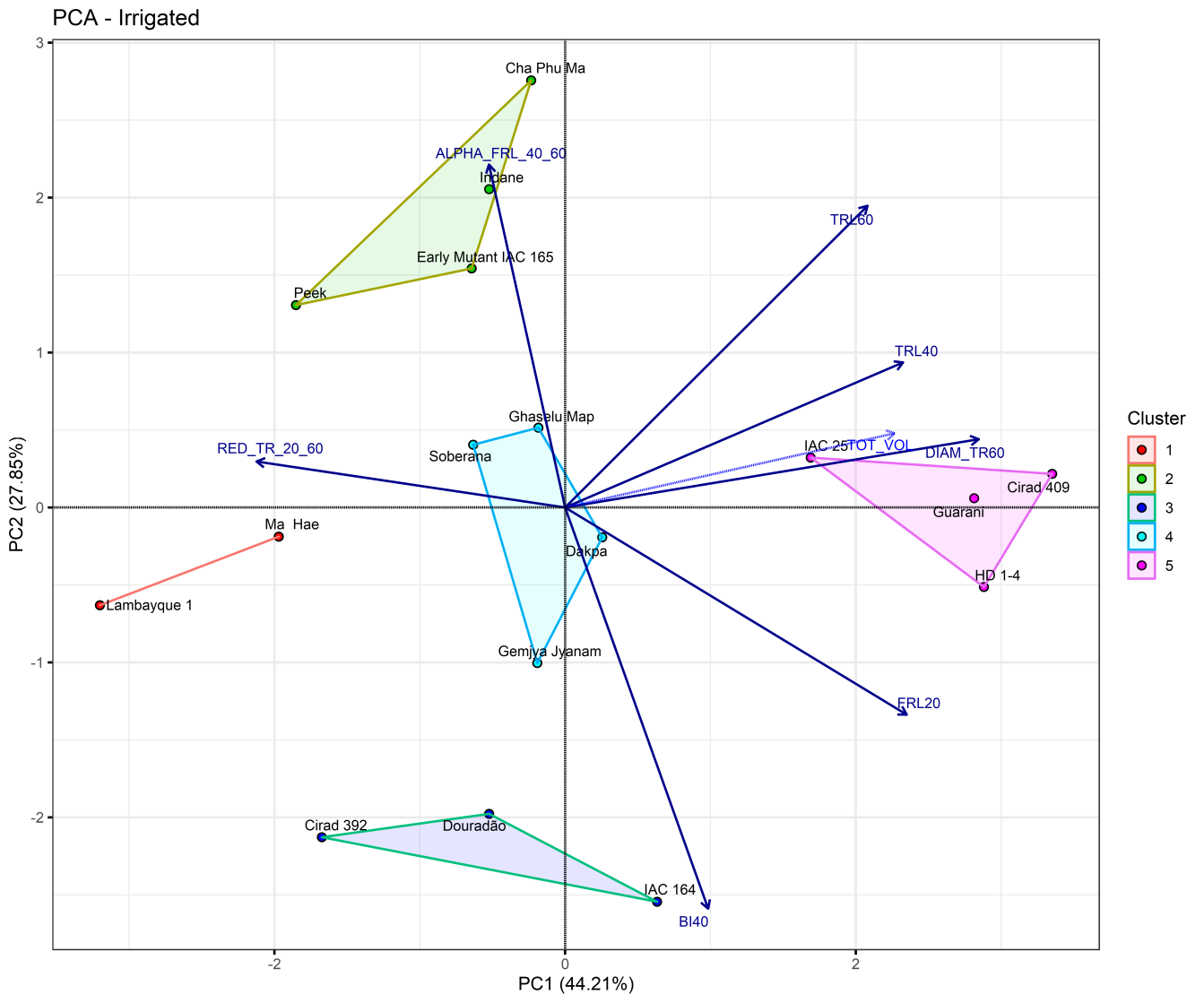
**

**Supplementary Fig. S3** Principal Component Analysis representation (plan 1-2) on the eight selected root traits among 17 rice accessions grown under irrigated conditions
