# Supplementary material for: Phenotyping Root Systems in a Set of Japonica Rice Accessions: Can Structural Traits Predict the Response to Drought?
Source: Rice (N Y). 2020 Sep 15;13:67. doi: 10.1186/s12284-020-00404-5 (PMC7492358; doi:10.1186/s12284-020-00404-5)
Supplement: Supplementary file 4 — Supplementary Fig. S4. Hierarchical Classification Analysis on the eight selected root traits among 17 rice accessions grown under irrigated conditions. The 17 rice accessions were assigned to one of the five general clusters (Cluster I, Cluster II, Cluster III, Cluster IV and Cluster V). The eight root traits were the same as those used for the PCA in Table 3. [file 12284_2020_404_MOESM4_ESM.docx]

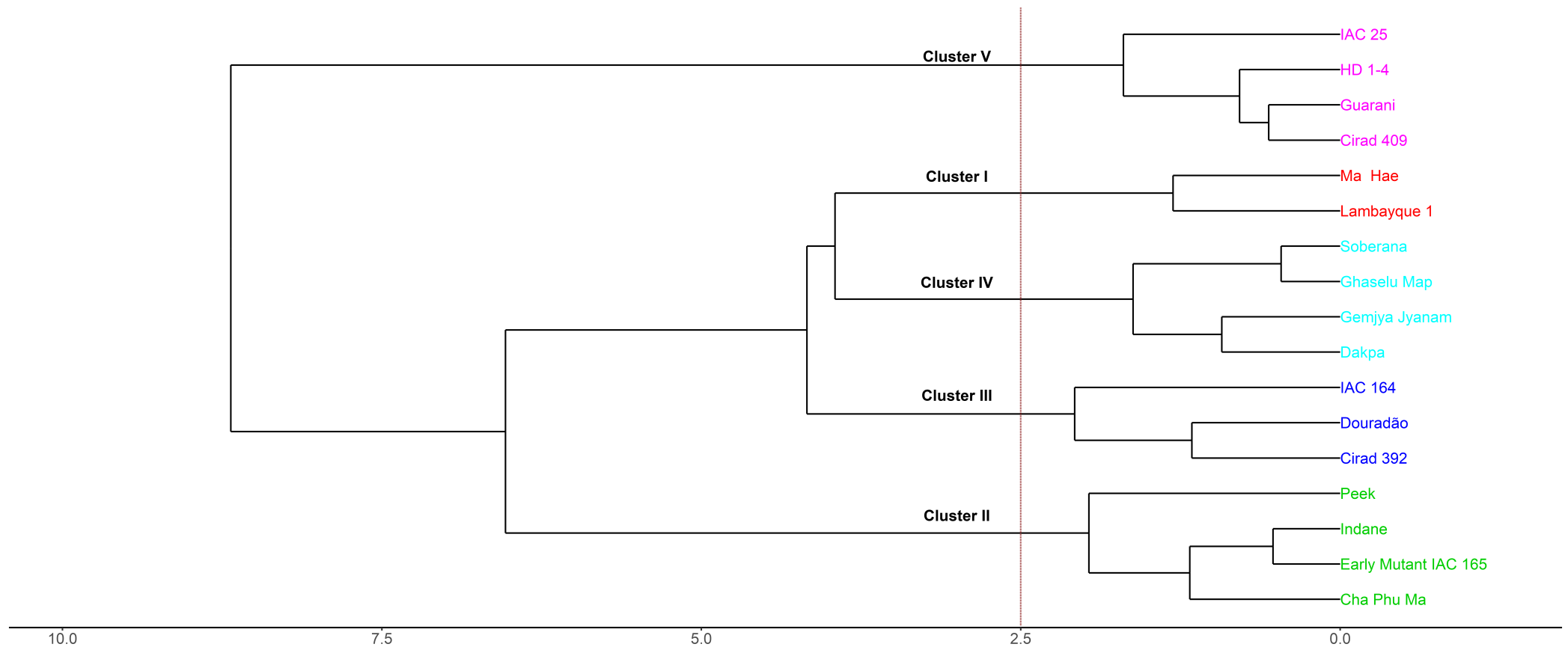


**Supplementary Fig. S4** Hierarchical Classification Analysis on the eight selected root traits among 17 rice accessions grown under irrigated conditions. *The 17 rice accessions were assigned to one of the five general clusters (Cluster I, Cluster II, Cluster III, Cluster IV and Cluster V). The eight root traits were the same as those used for the PCA in Table 3*
