# Supplementary material for: Phenotyping Root Systems in a Set of Japonica Rice Accessions: Can Structural Traits Predict the Response to Drought?
Source: Rice (N Y). 2020 Sep 15;13:67. doi: 10.1186/s12284-020-00404-5 (PMC7492358; doi:10.1186/s12284-020-00404-5)
Supplement: Supplementary file 5 — Supplementary Fig. S5. Radar chart for the different root system profiles under irrigated conditions. Each circle represents 20, 40, 60, 80 and 100% of variability found for each variable by the hierarchical cluster analysis (HCA). [file 12284_2020_404_MOESM5_ESM.docx]

**
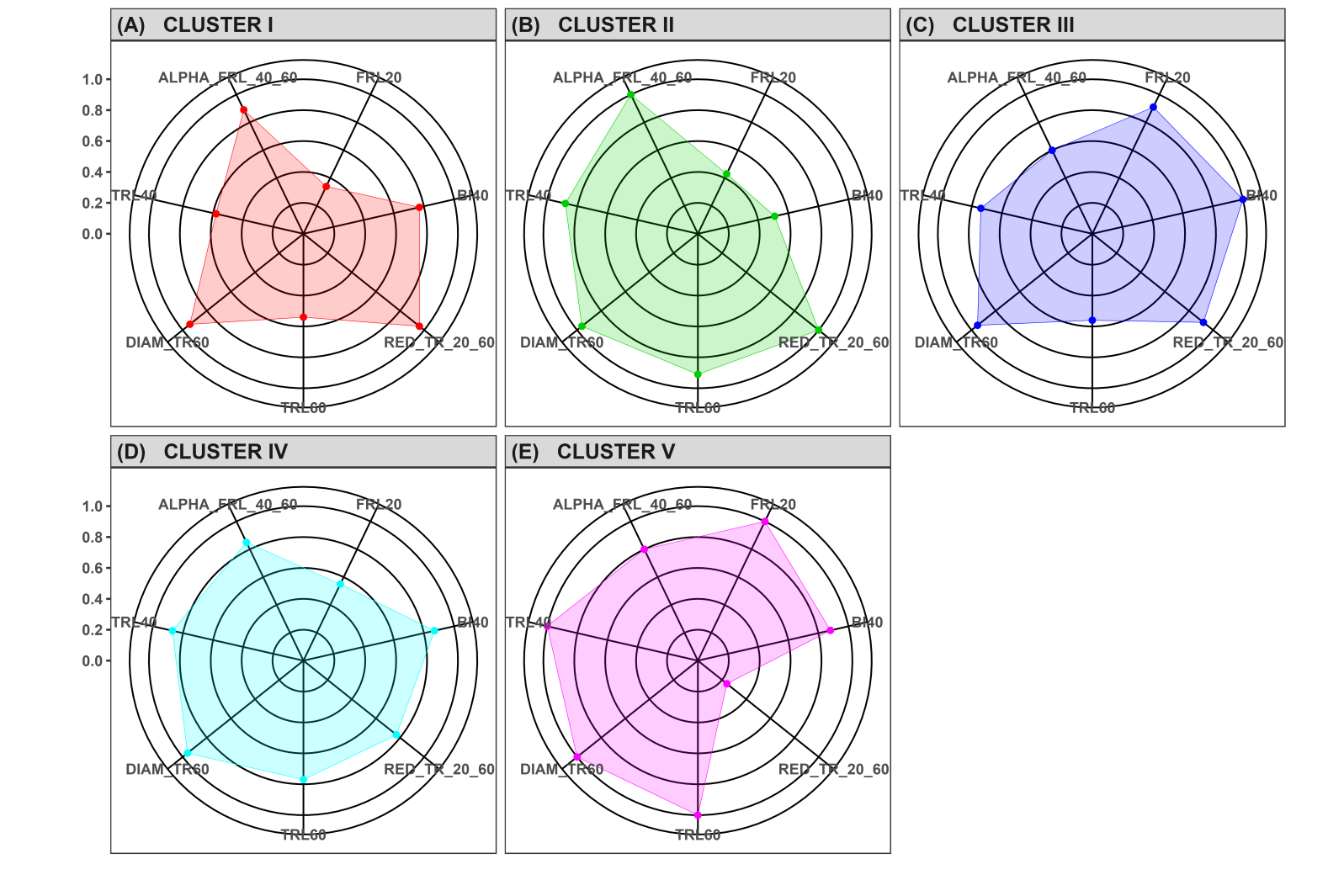
**

**Supplementary Fig. S5** Radar chart for the different root system profiles under irrigated conditions. *Each circle represents 20, 40, 60, 80 and 100% of variability found for each variable by the hierarchical cluster analysis (HCA)*
