# Supplementary material for: Phenotyping Root Systems in a Set of Japonica Rice Accessions: Can Structural Traits Predict the Response to Drought?
Source: Rice (N Y). 2020 Sep 15;13:67. doi: 10.1186/s12284-020-00404-5 (PMC7492358; doi:10.1186/s12284-020-00404-5)
Supplement: Supplementary file 6 — Supplementary Fig. S6 Cumulative root volume of the 17 accessions. Root volume was measured by 4 cm layers from 60 cm depth to soil surface. [file 12284_2020_404_MOESM6_ESM.docx]

**
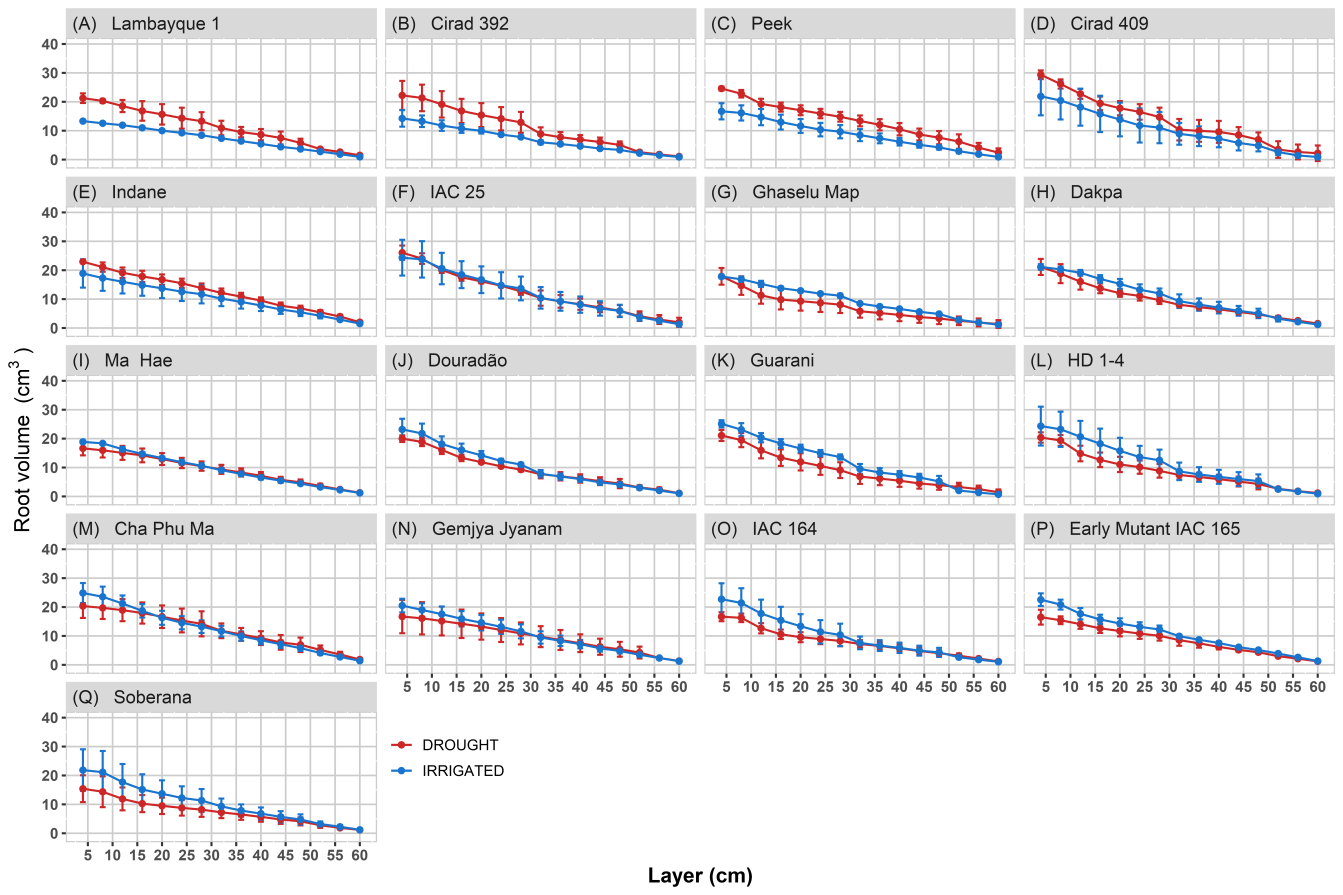
**

**Supplementary Fig. S6** Cumulative root volume of the 17 accessions. *Root volume was measured by 4 cm layers from 60 cm depth to soil surface*
