# Supplementary material for: Phenotyping Root Systems in a Set of Japonica Rice Accessions: Can Structural Traits Predict the Response to Drought?
Source: Rice (N Y). 2020 Sep 15;13:67. doi: 10.1186/s12284-020-00404-5 (PMC7492358; doi:10.1186/s12284-020-00404-5)
Supplement: Supplementary file 7 — Supplementary Fig. S7 Bar plots and standard error of the eight selected root traits for each accession under two water conditions. Fine root length (FRL20), thick root length (TRL40 and TRL60), branching index (BI40), thick root diameter (DIAM _ TR60), coefficient of maintenance for fine roots between 40 and 60 cm (${\alpha}_{FR{L}_{40\_60}}$αFRL40_60), thick root diameter reduction between 20 and 60 cm (${RED}_{T{R}_{20_{60}}}$REDTR2060) and total root volume (TOTVOL). Values are means of three replicates ± SD. [file 12284_2020_404_MOESM7_ESM.docx]

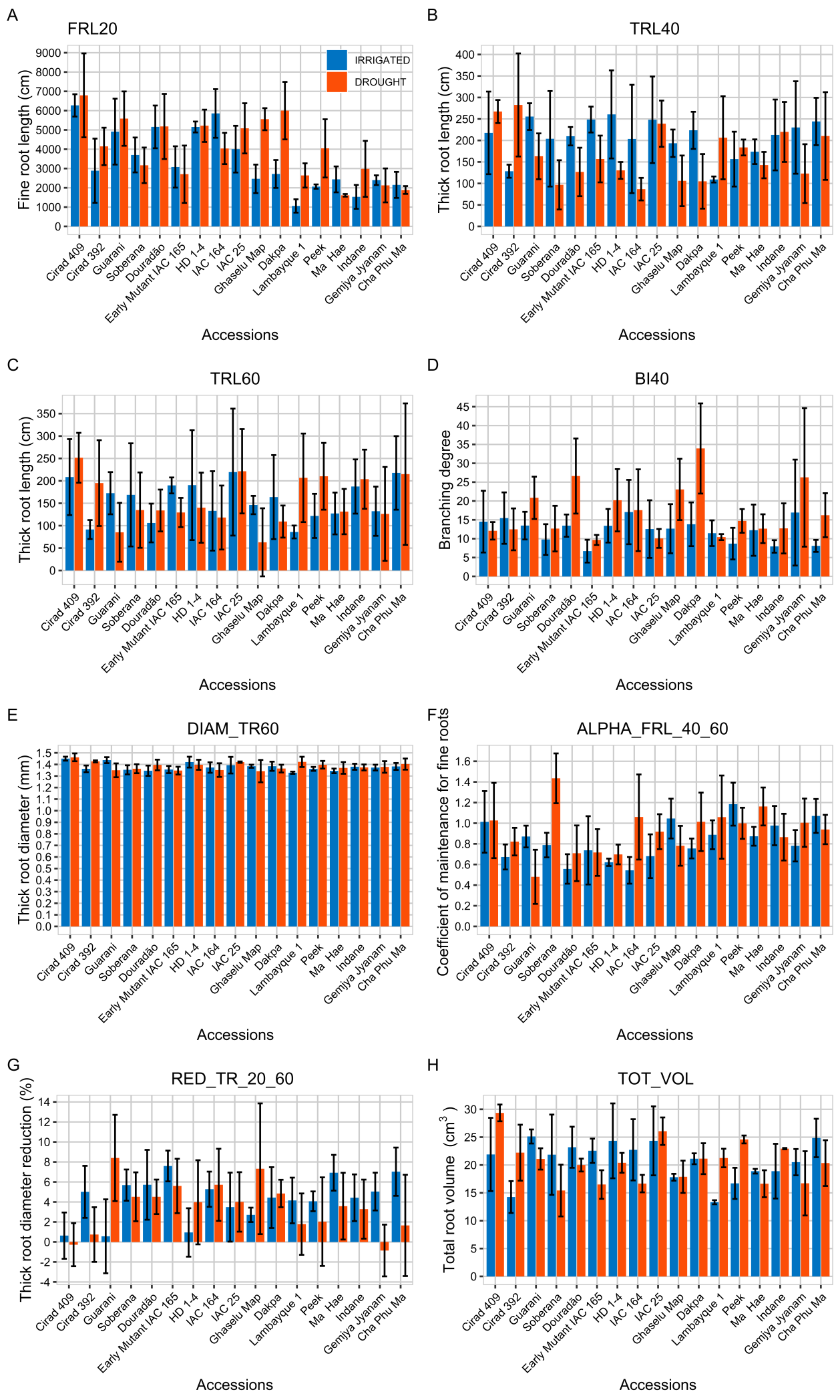


**Supplementary Fig. S7** Bar plots and standard error of the eight selected root traits for each accession under two water conditions. *Fine root length (*$FRL_{20}$*), thick root length (*$TRL_{40}$ *and* $TRL_{60}$*), branching index (*${BI}_{40}$*), thick root diameter (*${DIAM\_TR}_{60}$*), coefficient of maintenance for fine roots between 40 and 60 cm (*$\alpha_{FRL_{40\_60}}$*), thick root diameter reduction between 20 and 60 cm (*${RED}_{TR_{{20}_{60}}}$*) and total root volume (*${TOT}_{VOL}$*). Values are means of three replicates ± SD*
